# BAY11-7082 Targets RNF25 to Reverse TRIP4 Ubiquitination-dependent NF-κB Activation and Apoptosis Resistance in Renal Cell Carcinoma

**DOI:** 10.7150/ijbs.115032

**Published:** 2025-07-04

**Authors:** Lei Li, Zixi Wang, Bohan Ma, Qi Ye, Yuzeshi Lei, Mingming Lu, Leihong Ye, Jialu Kang, Wenyue Huang, Shan Xu, Ke Wang, Yule Chen, Jing Liu, Yang Gao, Chenji Wang, Jian Ma, Lei Li

**Affiliations:** 1 Department of Urology, The First Affiliated Hospital of Xi'an Jiaotong University, 710061, Xi'an, China.; 2 State Key Laboratory of Genetic Engineering, MOE Engineering Research Center of Gene Technology, Shanghai Engineering Research Center of Industrial Microorganisms, School of Life Sciences, Fudan University, Shanghai, China.

**Keywords:** anti-apoptosis, NF-κB activation, RNF25, TRIP4, BAY11-7082

## Abstract

NF-κB pathway dysregulation, a common driver of therapy resistance in cancer, promotes survival by suppressing apoptosis. While the anti-apoptotic role of NF-κB is recognized, the molecular mechanisms underlying this process remain poorly defined. Here, we identify the E3 ubiquitin ligase RNF25 as a key mediator of NF-κB-dependent apoptosis resistance in renal cell carcinoma cells, enabling evasion of multiple targeted therapies. Mechanistically, RNF25 binds TRIP4 and catalyzes its non-degradative ubiquitination at lysine 135, disrupting TRIP4-p65 interactions. This modification liberates p65 to activate NF-κB signaling, upregulating anti-apoptotic effectors (e.g., *cIAP2*, *Bcl-2*). We further demonstrate that the NF-κB inhibitor BAY11-7082 directly interacts with RNF25, reversing its pro-survival effects and restoring apoptosis sensitivity. Our findings establish RNF25 as a druggable orchestrator of therapy resistance through NF-κB pathway modulation and propose pharmacological targeting of RNF25 by BAY11-7082 as a strategy to overcome apoptosis resistance in renal malignancies.

## Introduction

Apoptosis, or programmed cell death, is a fundamental cellular process that maintains tissue homeostasis by eliminating damaged, unwanted, or potentially harmful cell 1-3. It is characterized by specific biochemical and morphological changes, such as cell shrinkage, DNA fragmentation, and membrane blebbing 4, all of which help to prevent the release of toxic cellular contents into the surrounding tissue. In cancer, however, the normal apoptotic pathways are often dysregulated, allowing malignant cells to evade death and proliferate uncontrollably 3, 5, 6. Evasion of apoptosis is a hallmark of cancer 7 and a major contributor to drug resistance, where cancer cells become unresponsive to chemotherapy and targeted therapies designed to induce apoptosis 6, 8, 9. Understanding the mechanisms through which tumor cells resist apoptosis is crucial for developing new therapeutic strategies to overcome drug resistance and enhance the effectiveness of cancer treatments 8, 10.

The nuclear factor kappa B (NF-κB) pathway is a critical signaling cascade that regulates immune responses, inflammation, cell survival, and apoptosis 11-13. Activated by various stimuli, including cytokines, growth factors, and stress signals, NF-κB translocates to the nucleus, where it controls the expression of genes involved in cell survival, proliferation, and inflammation 14, 15. NF-κB plays a dual role in cancer: it supports immune defense by enhancing cytotoxic immune cell activity against cancer cells, particularly during acute inflammation 16-18. Conversely, chronic NF-κB activation can drive pro-tumorigenic effects, as evidenced by the increased cancer risk in patients with chronic inflammatory diseases or those who are immune-suppressed 19, 20. Dysregulation of NF-κB promotes tumor growth, survival, and resistance to therapy by upregulating anti-apoptotic genes such as *Bcl-2*, *Bcl-xL*, and *XIAP*, thereby inhibiting apoptosis 21-25. These diverse roles make NF-κB a complex target in cancer therapy, highlighting the need to understand its context-specific functions, particularly in relation to apoptosis 26.

In the present study, we identify the E3 ubiquitin ligase RNF25 as a core regulator of apoptosis suppression in renal cell carcinoma via pathological NF-κB activation. RNF25 interacts with TRIP4 to catalyze site-specific ubiquitination at lysine 135, a non-proteolytic modification that prevents TRIP4-p65 complex assembly. This segregation unleashes p65-dependent NF-κB signaling, elevating survival proteins (e.g., *cIAP2*, *Bcl-2*) and imparting multidrug resistance. Significantly, we show that BAY11-7082—a known NF-κB inhibitor—directly targets RNF25, disrupting its survival network and restoring therapeutic susceptibility in resistant tumors.

## Materials and Methods

### Ethics statement and mice

All *in vivo* experiments were conducted in accordance with the Guidelines for the Ethical Review of Laboratory Animal Welfare (GB/T 35892-2018) and were approved by the Institutional Animal Care and Use Committee of Xi'an Jiaotong University (No. XJTULAC201). Before experimentation, the mice were acclimated for two weeks under standard housing conditions, including a 12h light/dark cycle, a temperature of 20-24°C, 40-60% humidity, 40-60% humidity, and a standard rodent chow diet.

### Cell culture

293T cells were cultured in DMEM medium (Biological Industries, #C3113-0500) supplemented with 10% FBS (Biological Industries, #04-001-1A) and 1% penicillin-streptomycin (NCM Biotech, #C125C5). HK-2, SW839, OS-RC-2, and H1299 cells were maintained in RPMI 1640 medium (Biological Industries, #C3010-0500) with the same supplements. Cells were routinely checked for mycoplasma contamination and returned negative results. The identity and purity of the cell lines were verified by the vendors using the short tandem repeat (STR) method.

### Establishment of apoptosis-resistant cell lines

SW839 and OS-RC-2 cells were cultured in RPMI 1640 medium supplemented with ABT-199 (Selleck, #S8048) in gradually increasing concentrations, starting from 1 µM and doubling every 1-2 weeks until reaching 400 µM over a period of 3 months. This process was performed to establish apoptosis-resistant cell lines, designated as SW839-R (SW839-resistant) and OS-RC-2-R (OS-RC-2-resistant) cells. After developing resistance, the cells were cultured in drug-free RPMI 1640 medium for 48 hours to eliminate residual ABT-199 before conducting downstream experiments. The resistance phenotype was confirmed by MTT assays, flow cytometry and protein expression analysis of apoptosis-regulating factors.

### Transfection and viral infection

Cells were transfected with indicated plasmids using PEI (Polysciences, #23966-2) or Lipofectamine 2000 (Invitrogen, #11668019) according to the manufacturer's instructions. For lentiviral infection, 293T cells were transfected with packaging vectors (pMD2.G and psPAX2) along with pLKO, RNF25 shRNA, p65 shRNA, or TRIP4 shRNA plasmids. The virus-containing supernatant was collected 48 hours post-transfection. To generate stable cell lines, target cancer cells were infected with the supernatant in the presence of polybrene (4 µg/ml) and selected in growth medium containing 1.5 µg/ml puromycin for at least three days. Cycloheximide (CHX) (MCE, #HY-12320) assays were performed as previously described 27.

### Western blot

Cell lysates were prepared by lysing cells in IP buffer (50 mM Tris-HCl, pH 7.4; 150 mM NaCl; 0.1% NP-40) containing protease and phosphatase inhibitors (Sigma-Aldrich), followed by centrifugation at 13,000 rpm for 15 minutes at 4°C. The supernatant was collected and protein concentration was quantified using a BCA protein assay (Invitrogen, #23225). Protein samples were prepared with 5x SDS loading buffer (250 mM Tris-HCl, pH 6.8; 10% SDS; 25 mM β-mercaptoethanol; 30% glycerol; 0.05% bromophenol blue) and boiled for 10 minutes. Equal amounts of protein were subjected to SDS-PAGE and transferred to nitrocellulose membranes. After blocking with 5% milk for 1 hour at room temperature, membranes were incubated with primary antibodies overnight at 4°C. The following day, membranes were washed three times with 1x TBST (20 mM Tris, 100 mM NaCl, 0.1% Tween-20) and incubated with secondary antibodies for 1 hour at room temperature. Protein bands were visualized using ECL Western blot substrate (Bio-Rad, #1705062). Relative protein expression was quantified using Image J software (version 1.53f51, NIH) by measuring the densitometry of the bands in the Western blot.

### Co-immunoprecipitation

The cells were transfected with specific plasmids and incubated for 48 hours. Afterward, they were harvested and lysed on ice for 15 minutes in IP buffer (50 mM Tris-HCl, pH 7.4; 150 mM NaCl; 0.1% NP-40) containing protease and phosphatase inhibitors. The cell lysate was then centrifuged at 13,000 rpm for 15 minutes at 4°C, and the supernatant was incubated overnight at 4°C with primary antibody-conjugated protein A/G beads (Invitrogen, #20422) or HA-/Flag-conjugated agarose beads (Sigma-Aldrich), with gentle rotation. The following day, the beads were washed at least four times with IP buffer on ice, and the proteins were eluted from the agarose beads by boiling at 95°C for 10 minutes. Finally, the proteins were subjected to Western blot analysis.

### *In vivo* ubiquitination assay

293T cells were transfected with the indicated plasmids and incubated for 48 hours. Cells were then harvested in denaturing buffer (6 M guanidine-HCl, 0.1 M Na2HPO4/NaH2PO4, and 10 mM imidazole). Ubiquitinated proteins were purified using Ni-NTA (Ni-nitrilotriacetic acid) resin (MCE, #HY-K0241), followed by Western blot analysis.

### *In vitro* ubiquitination assay

GST-TRIP4 and His-RNF25 proteins were expressed and purified in BL21 (DE3) E. coli. The *in vitro* ubiquitination reaction was carried out in a 30 μL reaction buffer consisting of 20 mM HEPES (pH 7.2), 5 mM MgCl₂, 0.1 mM DTT, and 1 mM ATP. The reaction included 50 nM E1 enzyme, 500 nM E2, and 1 μg of ubiquitin (Yeasen Bio, #20440ES10). To initiate the reaction, 500 ng of His-RNF25 (wild-type or 2CA mutant) and 500 ng of GST-TRIP4 were added and the reaction mixture was incubated at 30 °C for 2 hours. Reactions were terminated by adding 5× SDS-PAGE loading buffer, and the samples were resolved by SDS-PAGE followed by Western blot analysis with the indicated antibodies.

### Total RNA extraction and quantitative real-time polymerase chain reaction (qRT-PCR) analysis

Total RNA was isolated from cells using TRIzol (Invitrogen, #15596018CN). cDNA was synthesized using PrimeScript RT Master Mix (TAKARA, #RR036A) according to the manufacturer's instructions. Quantitative real-time PCR was performed using 2× TSINGKE® Master qPCR Mix (SYBR Green I), with technical triplicates and biological replicates, on a CFX96 detection system (Bio-Rad). Gene expression was normalized to *18S rRNA*, and quantitative analysis was conducted using the 2-ΔΔCt method. Primer sequences are listed in Table S1.

### Detection of apoptosis using annexin V assay and flow cytometry

Cells were washed twice with cold PBS and resuspended in 1× Binding Buffer. A total of 1 × 10⁵ cells were stained with PE Annexin V and 7-amino-actinomycin D (7-AAD) according to the manufacturer's instructions for the PE Annexin V Apoptosis Detection Kit I (BD Biosciences, #559763). The cells were incubated at room temperature for 15 minutes and then analyzed using a flow cytometer. Data analysis was performed with FlowJo software (version 10.8.1, FlowJo, LLC).

### Dual luciferase reporter assay

Control and knockdown cells were seeded in a 6-well plate. The NF-κB response element reporter (pGL3- NF-κB) (MiaoLing Biology, #P6708) was co-transfected with the Renilla reporter (pRL-TK) (MiaoLing Biology, #P0372) using Lipofectamine 2000. Cells were lysed with passive lysis buffer (Promega, #E1941), and relative luciferase activity was assessed by measuring firefly luciferase activity and normalizing it to Renilla luciferase activity, following the manufacturer's instructions for the Dual-Glo Luciferase Assay System (Promega, #E2920).

### Cell viability assay

Cells were seeded into 96-well plates at a density of 3,000 cells per well and cultured in 100 µl of the indicated medium containing 10% serum. After 24 hours, cells were treated with various concentrations of compounds or left untreated in 100 µl of medium for an additional 48 hours. Cell viability was assessed using methyl thiazol tetrazolium (MTT) (Sigma-Aldrich, #475989) according to the manufacturer's instructions. Absorbance at 570 nm was measured using an Epoch Microplate Spectrophotometer (BioTek). All cell viability experiments were conducted in triplicate, and the average represents one biological replicate.

### Colony formation assay

Cells were seeded into six-well plates at a density of 1,000 cells per well in the indicated medium and cultured for 1 to 2 weeks, depending on colony size. Following incubation, cells were fixed with 4% paraformaldehyde for 15 minutes, stained with 0.5% w/v crystal violet for 30 minutes. The colonies were then gently washed with running water and photographed. Colonies containing more than 50 cells were counted using ImageJ software (version 1.53f51, NIH).

### Mass spectrometry analysis

Liquid chromatography-mass spectrometry (LC-MS) analysis was performed as previously described 28. For the analysis of the RNF25 and p65 interactomes, 293T cells were transfected with Flag-tagged RNF25 or HA-tagged p65 and incubated for 48 hours. The cells were then lysed in IP buffer, and immunoprecipitation was performed using Flag- or HA-conjugated agarose beads (Sigma-Aldrich). Bound proteins were eluted with 2% SDS and digested overnight with sequencing-grade modified trypsin (Promega, #PRV5111). The resulting peptides were analyzed using a nanoflow EASY-nLC 1200 system (Thermo Fisher Scientific, Odense, Denmark) coupled to an Orbitrap Exploris 480 mass spectrometer (Thermo Fisher Scientific, Bremen, Germany). Data were processed using the UniProt human protein database (75,004 entries, downloaded on 07-01-2020) with Protein Discoverer (version 2.4.1.15, Thermo Fisher Scientific) and Mascot (version 2.7.0, Matrix Science).

For TRIP4 ubiquitination site mapping, 293T cells were transfected with Flag-TRIP4, HA-Ub, and Myc-RNF25. The co-IP sample was then analyzed via LC-MS using a nanoflow EASY-nLC 1000 system (Thermo Fisher Scientific, Odense, Denmark) coupled to an Orbitrap Elite mass spectrometer (Thermo Fisher Scientific, Bremen, Germany). Results were processed using the UniProt human protein database (70,956 entries, downloaded on 12-02-2016) with Protein Discoverer (version 1.4.0.288, Thermo Fisher Scientific) and Mascot (version 2.3.2, Matrix Science). Identified TRIP4 ubiquitination sites were validated through mutagenesis and co-IP assays. All mass spectrometry experiments were performed once.

### Mass spectrometry-based proteomics analysis

Proteins from both parental and resistant cells were extracted from biological samples using IP buffer containing 100 mM DTT, 50 mM iodoacetamide (IAM), and protease inhibitors. Protein concentrations were measured using a BCA assay (Invitrogen, #23225), followed by overnight digestion with trypsin (1:30) at 37°C. The resulting peptides were analyzed using the synchronous precursor selection (SPS)-MS3 method on an Orbitrap Fusion Lumos Tribrid mass spectrometer (Thermo Fisher Scientific, Bremen, Germany). For protein identification, the raw data were processed using the UniProt human protein database (75,004 entries, downloaded on 07-01-2020) with Protein Discoverer (version 2.4.1.15, Thermo Fisher Scientific). For protein quantification, the top three unique and razor peptides with a reporter ion mass tolerance of less than 20 ppm were selected. Normalization was performed against the total peptide amount.

### RNA-seq and bioinformatic analysis

Total RNA was extracted from control and RNF25-knockdown SW839 cells using TRIzol reagent (Invitrogen, #15596018CN) following the manufacturer's guidelines. High-quality total RNA (RIN > 7.0, as determined by Agilent Bioanalyzer) was used to prepare sequencing libraries with the Illumina TruSeq Stranded Total RNA/Ribo-Zero Sample Prep Kit. A total of 500-1,000 ng of riboRNA-depleted total RNA was fragmented with RNase III at 37°C for 10-18 minutes, after which RNase III was inactivated at 65°C for 10 minutes. Size selection for 50- to 150-bp fragments was performed using the FlashPAGE denaturing PAGE-fractionator (Thermo Fisher Scientific), followed by overnight ethanol precipitation. The resulting RNA was directionally ligated, reverse-transcribed, and treated with RNase H. Differential expression analysis was conducted using the DESeq2 (v1.30.1) Bioconductor package. Genes were considered differentially expressed if they met the criteria of a P value < 0.05, an adjusted P value < 0.05, and a fold change ≥ 1.5, with adjustments made using Benjamini and Hochberg's method to control the false discovery rate.

### Nuclear-cytoplasmic fractionation

Trypsin-dispersed cells were neutralized and collected by centrifugation at 1500 × g using a microcentrifuge. The cell pellet was gently resuspended in PBS. One-third of the resuspended cell volume was used for immunoblot analysis of whole-cell lysates as described above. The remaining two-thirds were centrifuged again, and the pellet was resuspended in low-salt buffer (20 mM HEPES, pH 7.9; 1.5 mM MgCl₂; 20 mM KCl; 0.2 mM EDTA; 5% glycerol; 0.01% (v/v) NP-40; 0.5 mM DTT; and protease inhibitors). The cells were incubated on ice for 25 minutes with occasional gentle flicking to lyse the cytoplasmic membrane. Following incubation, nuclei were pelleted by centrifugation at 1500 × g for 15 minutes. The supernatant, representing the cytoplasmic fraction, was carefully transferred to a clean microcentrifuge tube.

The nuclear pellet was washed twice with low-salt buffer and centrifuged again at 1500 × g for 15 minutes. The resulting nuclear pellet was then resuspended in high-salt buffer (20 mM HEPES, pH 7.9; 1.5 mM MgCl₂; 420 mM KCl; 0.2 mM EDTA; 5% glycerol; 0.5 mM DTT; and protease inhibitors). Nuclear lysates were vortexed briefly and sonicated for 1 minute using an ultrasonic cell disruptor. The samples were then incubated with gentle rotation for 40 minutes at 4°C. Both the cytoplasmic and nuclear fractions were centrifuged at 12,000 rpm for 10 minutes to pellet any remaining nuclei or debris. The resulting supernatants were collected separately in clean tubes and subjected to immunoblotting.

### Chromatin fractionation assay

Cells were washed with PBS and lysed in cell surface kinase (CSK) buffer (10 mM HEPES, pH 7.9; 100 mM NaCl; 300 mM sucrose; 0.1% Triton X-100; and protease inhibitors) on ice for 20 minutes. Following lysis, the samples were centrifuged at 12,000 rpm for 10 minutes, and the total lysate was collected. The pellet obtained from this centrifugation step was washed twice with CSK buffer and then resuspended in a solution containing deoxyribonuclease I and 300 mM NaCl. The suspension was incubated at 25°C for 30 minutes. After sonication, the resulting lysate represented the chromatin fraction.

### Immunofluorescent analysis and quantification

SW839 or OS-RC-2 cells grown on 13- mm glass coverslips were fixed with 4% formaldehyde for 15 min, then permeabilized with 0.2% Triton X-100 for 10 minutes at room temperature. The coverslips were subsequently blocked with 5% BSA for 1 hour. Primary antibodies were applied and incubated overnight at 4°C. The following day, the coverslips were washed at least three times with PBS containing 0.01% Tween-20 (PBS-T) and then incubated with fluorophore-conjugated secondary antibodies for 1 hour at room temperature. After three additional washes, the coverslips were mounted onto glass slides using mounting medium containing 4',6-diamidino-2-phenylindole (DAPI). Fluorescence images were acquired using a confocal microscope. The nuclear-to-cytoplasmic ratio of p65 fluorescence intensity in each cell was quantified using ImageJ software (version 1.53f51, NIH). Approximately 50 cells were analyzed per experimental condition.

### Chromatin Immunoprecipitation Sequencing (ChIP-seq)

SW839 cells with TRIP4 knockdown were fixed with 1% formaldehyde, followed by quenching with 0.125 mM glycine to stop the fixation. To obtain 200-800 bp chromatin fragments, sonication was performed using the Covaris S220. The lysate was then cleared by centrifugation at 500g for 5 minutes at 4°C and incubated overnight at 4°C with an anti-p65 antibody (sc-8008X, Santa Cruz Biotechnology), ChIP-Grade Protein G Magnetic Beads (9006, Cell Signaling Technology), and 1% Triton X-100 (Roche, cat# 10789704001). All buffers used in the steps above were supplemented with protease inhibitors (Roche, cat# 11697498001).

The next day, antibody-chromatin complexes captured on beads were sequentially washed with 1× RIPA buffer (140 mM NaCl, 1 mM EDTA, 10 mM Tris-HCl pH 8.0, 1% Triton X-100, 0.1% SDS, 0.1% sodium deoxycholate, and 1× protease inhibitor cocktail), 1× RIPA buffer + 0.5 M NaCl, LiCl Wash Buffer, and TE Buffer before elution. To elute the p65-bound chromatin, samples were treated with RNase (Roche, cat# 10109169001) and Proteinase K (Invitrogen, cat# 25530-049), followed by reverse crosslinking at 65°C overnight. DNA was then purified using the QIAquick PCR Purification Kit (Qiagen, cat# 28106).

Libraries were prepared using the NEBNext Ultra II DNA Library Prep Kit for Illumina (NEB, cat# E7645S), incorporating single (NEB, cat# E7335, cat# E7710) or dual (NEB, cat# E7600, cat# E7780) indexing. Two replicates were performed for each condition. Indexed libraries were validated for quality and size distribution using a TapeStation 4150 (Agilent), quantified with the KAPA Library Quantification Kit (Roche, cat# KK4824), and paired-end sequenced (38 bp + 38 bp) on an Illumina NextSeq 550.

### Isothermal titration calorimetry (ITC) assay

RNF25 protein was expressed in BL21 *E. coli* and purified via affinity chromatography. BAY11-7082 was dissolved in DMSO. Prior to ITC experiments, the sample cell and injection syringe were cleaned according to the manufacturer's protocol. The sample cell was rinsed multiple times with buffer and loaded with 0.2 ml of protein solution (~20 μM), ensuring no bubbles formed. The syringe was filled with 0.04 ml of peptide solution (~200 μM). ITC experiments were conducted at 25°C, starting with a small initial injection (0.4 μl), followed by 19 injections of 2 μL each. After the experiment, ITC data were analyzed using the MicroCal iTC200 system (Malvern Panalytical, United Kingdom) and MicroCal PEAQ-ITC Analysis Software (version 1.41, Malvern Panalytical).

### Generation and treatment of kidney cancer xenografts in mice

SW839 cells, with or without RNF25 overexpression (1 × 10^6^), were resuspended in serum-free medium and mixed with Matrigel (Corning, #354234) at a 1:1 ratio before being injected into the right flank of male nude mice. The mice were randomly assigned to groups, with ten mice per group. BAY 11-7082 (10 mg/kg) was administered via intraperitoneal injection, and axitinib (30 mg/kg) was given via oral gavage, with both treatments lasting for 14 days, starting when the tumor volume reached 100-150 mm³. Tumor size was measured every 5 days using calipers, and tumor volume was calculated using the formula: Volume (mm³) = 0.5 × (length) × (width)², where length is the longest diameter and width is the shortest diameter. Mice were sacrificed when the tumor volume reached the predetermined endpoint or when they were deemed moribund. The animal experiments were conducted according to protocols approved by the Rules for Animal Experiments published by the Chinese Government and approved by the Institutional Animal Care and Use Committee of Xi'an Jiaotong University.

### Quantification and statistical analysis

All graphs were generated using GraphPad Prism 9 (version 9.1.2, GraphPad, Inc). Differences between groups were analyzed by two-sided unpaired Student's *t*-test, one-way ANOVA or two-way ANOVA with Microsoft Office Excel 2010 or GraphPad Prism 9.

## Results

### RNF25 drives apoptosis suppression and mediates resistance to diverse targeted therapies

**Figure 1:**
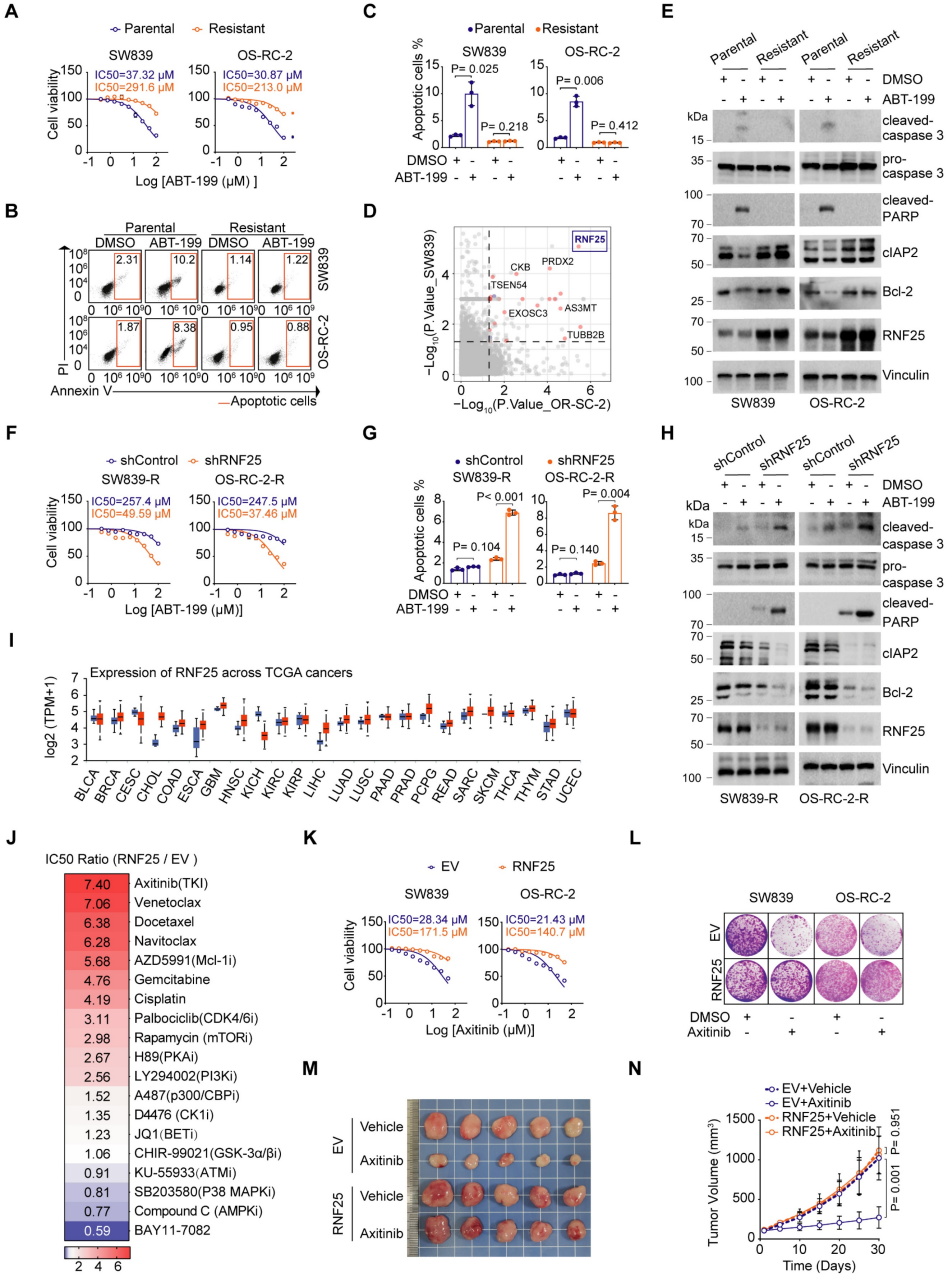
** RNF25 drives apoptosis suppression and mediates resistance to diverse targeted therapies. A** Dose-response survival curves of parental and resistant SW839 (**left**), and OS-RC-2 (**right**) cells exposed to increasing concentrations of ABT-199 (mean ± SEM, n = 3). **B, C** Annexin V/7-AAD-Flow cytometry (FC) analysis of parental and resistant SW839 and OS-RC-2 cells treated with DMSO or ABT-199 (20 µM) for 24 hours (**B**), with quantification data shown in (**C**) (mean ± SD, n = 3, two-tailed unpaired Student's *t*-test). **D** Differentially expressed proteins between parental and resistant cells. Top screening hits are highlighted in red. **E** Western blot analysis of whole-cell lysis (WCL) derived from parental and resistant SW839 and OS-RC-2 cells treated with DMSO or ABT-199 (20 µM) for 24 hours. **F** Dose-response survival curves of control and RNF25-knockdown SW839-R (**left**) and OS-RC-2-R (**right**) cells exposed to increasing concentrations of ABT-199 (mean ± SEM, n = 3). **G** Apoptosis quantification of control and RNF25-knockdown SW839-R and OS-RC-2-R cells treated with DMSO or ABT-199 was performed. The apoptotic cells were calculated as mean ± SD (n=3, two-tailed unpaired Student's *t*-test). **H** Western blot analysis of WCL derived from control and RNF25-knockdown SW839-R and OS-RC-2-R cells treated with DMSO or ABT-199 (20 µM) for 24 hours. **I** RNF25 expression across various cancer types and adjacent normal tissues from TCGA is shown in a boxplot, sourced from UALCAN, with red representing tumor tissue and blue representing normal tissue.** J** Control and RNF25-overexpressing HK-2 were treated with various chemicals, and cell viability was measured by MTT assays. Heatmap showing the IC50 ratio. **K** Dose-response survival curves of control and RNF25-overexpressing SW839 (**left**) and OS-RC-2 (**right**) cells exposed to increasing concentrations of axitinib (mean ± SEM, n = 3). **L** Colony formation assays were performed in control and RNF25-overexpressing SW839 and OS-RC-2 cells treated with DMSO or axitinib (5 µM). **M, N** Control and RNF25-overexpressing SW839 cells were injected subcutaneously into the right flank of nude mice. Mice received oral gavage of either vehicle or axitinib (30 mg/kg) daily for 14 days. Tumors were harvested and photographed on day 30 (**M**). Tumor volume was measured at the indicated time points (**N**). (mean ± SD, n = 5, two-sided two-way ANOVA, n = 5). All experiments were independently performed in triplicate, yielding consistent results. Key abbreviations: P, parental; R, resistant; EV, empty vector.

Evasion of apoptosis is a key factor contributing to drug resistance in cancer treatment 29, 30. To explore the potential mechanisms behind apoptosis-mediated resistance in renal cell carcinoma (RCC), we developed apoptosis-resistant SW839 and OS-RC-2 RCC cell lines through continuous exposure to the selective Bcl-2 inhibitor ABT-199, which is commonly used in clinical settings to induce apoptosis, particularly in chronic lymphocytic leukemia (CLL) and non-Hodgkin lymphoma (NHL) 31 (Figure 1A-C). Mass spectrometry (MS)-based proteomics revealed an upregulation of several proteins associated with anti-apoptotic pathways, such as CKB 32, PRDX2 33, and TUBB2B 34, in apoptosis-resistant cell lines (Figure 1D). Among these, the E3 ligase RNF25 was notably increased in the resistant cells. Analysis of cell lysates from both parental and resistant cells further confirmed that ABT-199 treatment led to increased cleavage of caspase 3 and PARP in the parental cells, while the anti-apoptotic proteins cIAP2, along with RNF25, were elevated in the resistant cells (Figure 1E). Indeed, overexpression of RNF25 in primary SW839 and OS-RC-2 cells resulted in similar outcomes with the resistant cells (Figure S1A-E). Moreover, knockdown of RNF25 restored ABT-199-induced apoptosis in SW839 and OS-RC-2 resistant cells, accompanied by reduced expression of cIAP2 and Bcl-2 (Figure 1F-H, S1F and G), indicating that RNF25 functions as a negative regulator of apoptosis.

We further analyzed RNF25 expression levels across various cancer types using The Cancer Genome Atlas (TCGA) datasets and found that RNF25 was elevated in most cancers (Figure 1I). To simulate patient conditions, we stably overexpressed RNF25 in HK-2 normal kidney cells and treated them with a range of small molecule inhibitors targeting various cancer pathways. Notably, RNF25 overexpression significantly contributed to drug resistance, particularly to apoptosis-inducing agents like gemcitabine and docetaxel 35-38 (Figure 1J). Moreover, RNF25 overexpression conferred substantial resistance to axitinib, a widely used tyrosine kinase inhibitor for advanced renal cell carcinoma 39-41. Dose survival assays and colony formation in SW839 and OS-RC-2 cells corroborated these findings in both axitinib and two additional commercially available tyrosine kinase inhibitors, sorafenib and erlotinib (Figure 1K and L, S2A-G). *In vivo*, RNF25 overexpression diminished the efficacy of axitinib in inhibiting SW839 tumor growth (Figure 1M and N). Western blot analysis and TUNEL assays showed that axitinib treatment induced a higher level of tumor cell apoptosis in control tumors but not in those with high RNF25 expression (Figure S2H-J), indicating that elevated RNF25 levels promote axitinib resistance through anti-apoptotic mechanisms.

### NF-κB activation is necessary for RNF25-mediated anti-apoptosis

**Figure 2:**
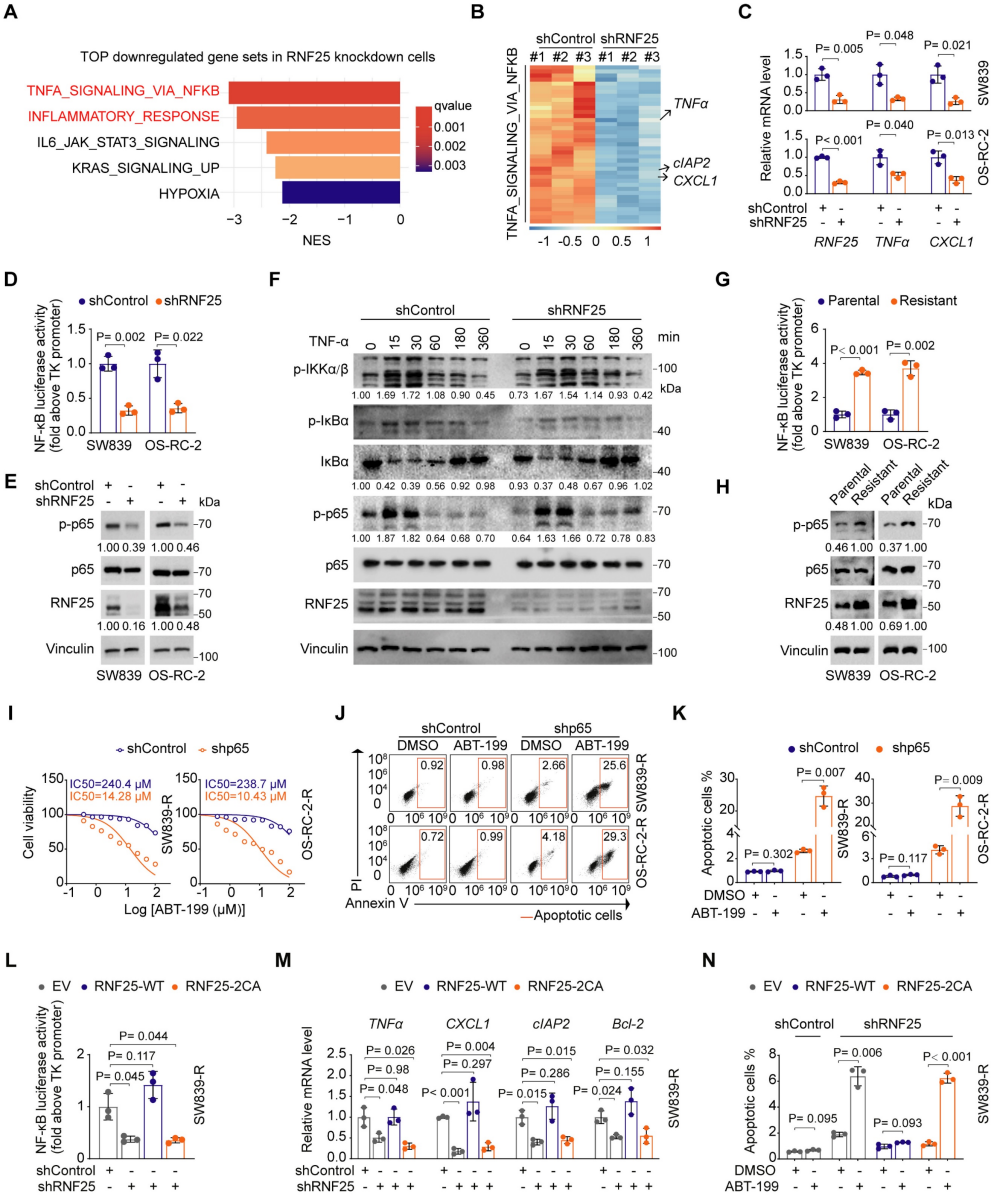
**NF-κB activation is necessary for RNF25-mediated anti-apoptosis. A** Top five enriched pathways associated with downregulated genes following RNF25 knockdown. **B** Heatmap showing the relative expression of NF-κB pathway genes significantly regulated by RNF25 knockdown (log2FC > 1, q-value < 0.05). **C, D** RT‒qPCR (**C**) and dual luciferase reporter assay (**D**) were performed in control and RNF25-knockdown SW839 and OS-RC-2 cells (mean ± SD, n = 3, two-tailed unpaired Student's *t*-test). **E** Western blot analysis of WCL derived from control and RNF25-knockdown SW839 and OS-RC-2 cells. Relative protein levels of RNF25 and p-p65 are shown. **F** Western blot analysis of WCL derived from control and RNF25-knockdown SW839 cells treated with 50 ng/ml TNFα for the indicated time points. Relative protein levels of p-IKKα/β, IκBα and p-p65 are shown. **G** Western blot analysis of WCL derived from parental and resistant SW839 and OS-RC-2 cells. Relative protein levels of RNF25 and p-p65 are shown. **H** Dual luciferase reporter assay were performed in parental and resistant SW839 and OS-RC-2 cells (mean ± SD, n = 3, two-tailed unpaired Student's *t*-test). **I** Dose-response survival curves of control and p65-knockdown SW839-R (**left**) and OS-RC-2-R (**right**) cells exposed to increasing concentrations of ABT-199 (mean ± SEM, n = 3). **J, K** Annexin V/7-AAD-FC analysis of control and p65-knockdown SW839-R and OS-RC-2-R cells treated with DMSO or ABT-199 (20 µM) for 24 hours (**J**), with quantification data shown in (**K**) (mean ± SD, n = 3, two-tailed unpaired Student's *t*-test). **L, M** Dual luciferase reporter assay (**L**) and RT‒qPCR (**M**) were performed in control and RNF25-knockdown SW839-R cells infected with the indicated viral constructs (mean ± SD, n = 3, two-tailed unpaired Student's *t*-test). **N** Quantification of Annexin V/7-AAD-FC analysis of control and RNF25-knockdown SW839-R cells infected with the indicated viral constructs and treated with DMSO or ABT-199 (20 µM) for 24 hours (mean ± SD, n = 3, two-tailed unpaired Student's *t*-test). All experiments were independently performed in triplicate, yielding consistent results.

To determine how RNF25 regulates apoptosis, we performed RNA-seq analysis on RNF25-knockdown SW839 cells. Gene set enrichment analysis (GSEA) revealed that NF-κB-related pathways, including TNF-α signaling and inflammatory responses, were among the most significantly downregulated pathways in RNF25 knockdown cells (Figure 2A).

Since NF-κB signaling is known to inhibit apoptosis 42, 43, we hypothesized that RNF25 may regulate apoptosis via the NF-κB pathway. Differential expression analysis confirmed that most NF-κB target genes were downregulated following RNF25 knockdown in SW839 cells (Figure 2B). Among them, key targets such as *TNFα, CXCL1*, and *cIAP2* 14 showed significant reductions, which were further validated by RT-qPCR (Figure 2C). Consistently, luciferase reporter assays demonstrated that RNF25 knockdown suppressed NF-κB activation (Figure 2D). Notably, we observed a moderate decrease in p65 phosphorylation under basal (unstimulated) conditions (Figure 2E), a key event required for NF-κB activation 44, 45. However, RNF25 knockdown did not affect TNF-α-induced phosphorylation of IκBα or IKKα/β (Figure 2F), indicating that RNF25 regulates NF-κB activity independently of the activation of upstream kinases. Besides, phosphorylation of p65 and overall NF-κB activity were markedly elevated in apoptosis-resistant SW839 and OS-RC-2 cells, supporting the hypothesis that RNF25 may regulate apoptosis through the NF-κB pathway (Figure 2G and H).

To further investigate the role of NF-κB activation in RNF25-mediated resistance to apoptosis, we knocked down p65 in the apoptosis-resistant SW839 and OS-RC-2 cells. This knockdown reduced the IC50 value for ABT-199 in these cells and restored apoptosis (Figure 2I-K). Similarly, silencing p65 in cells with high RNF25 expression led to a decrease in the IC50 value for ABT-199 and increased apoptosis compared to control cells (Figure S3A-C). Given that RNF25 functions as an E3 ubiquitin ligase 46, we investigated whether its ligase activity is required for NF-κB activation. We observed that reintroducing wild-type (WT) RNF25, but not the catalytically inactive 2CA mutant (C135A/C138A) 47, reversed the effects of RNF25 knockdown, restoring NF-κB activity and anti-apoptosis in apoptosis-resistant SW839 and OS-RC-2 cells (Figure 2L-N, S3D-I), indicating that the E3 ligase activity of RNF25 is essential for its regulation of NF-κB activation and anti-apoptotic function.

### TRIP4 is a ubiquitination target of RNF25 essential for NF-κB activation

**Figure 3:**
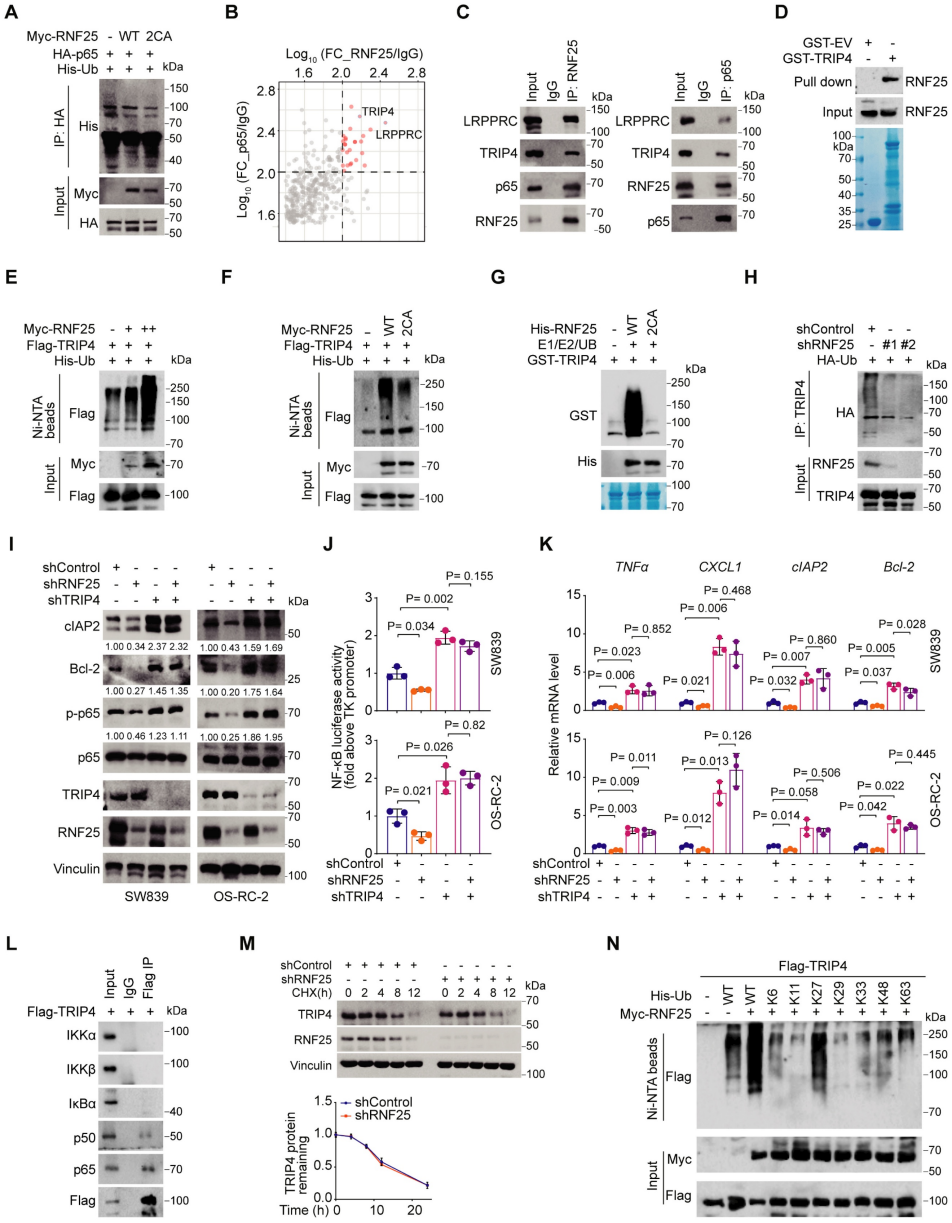
**TRIP4 is a ubiquitination target of RNF25 essential for NF-κB activation. A** Western blot analysis of input samples and immunoprecipitates (IP) derived from 293T cells transfected with HA-p65, His-ubiquitin (UB) and the indicated Myc-RNF25 constructs. **B** IP-MS ratio plot shows normalized Log_10_(FC_p65/RB1) versus Log_10_ (FC_RNF25/IgG), with co-interacting proteins highlighted in pink. **C** Western blot analysis of input samples and anti-RNF25 or anti-p65 immunoprecipitates derived from SW839 cells.** D** Western blot analysis of RNF25 proteins in SW839 cells pulled down by GST-EV or GST-TRIP4 recombinant proteins.** E** Western blot analysis of input samples and Ni-NTA (Ni-nitrilotriacetic acid) affinity precipitates from 293T cells transfected with Flag-TRIP4, His-UB, and increasing amounts of Myc-RNF25.** F** Western blot analysis of input samples and Ni-NTA affinity precipitates from 293T cells transfected with Flag-TRIP4, His-UB, and the indicated Myc-RNF25 constructs. **G** Wild-type (WT) and 2CA mutant His-RNF25 proteins were incubated with GST-TRIP4, along with E1, E2, and ubiquitin, at 30 °C for 60 minutes, followed by SDS-PAGE and Western blot analysis. **H** Western blot analysis of input samples and anti-TRIP4 immunoprecipitates derived from SW839 cells transfected with HA-UB.** I** Western blot analysis of WCL derived from SW839 and OS-RC-2 cells expressing indicated lentivirus-delivered shRNAs. Relative protein levels of p-p65, Bcl-2 and cIAP2 are shown. **J**,** K** Dual luciferase reporter assay (**J**) and RT‒qPCR (**K**) were performed in SW839 (**top**) and OS-RC-2 (**bottom**) cells expressing indicated lentivirus-delivered shRNAs (mean ± SEM, n = 3, two-tailed unpaired Student's *t*-test). **L** Western blot analysis of input samples and anti-Flag-TRIP4 immunoprecipitates derived from 293T cells. **M** Western blot analysis of WCL derived from control and RNF25-knockdown SW839 cells treated with 200 µg/µl CHX at the indicated time points (**top**), and protein bands were quantified (**bottom**). **N** Western blot analysis of WCL and Ni-NTA affinity precipitates derived from 293T cells transfected with Flag-TRIP4 and the indicated Myc-RNF25, WT, or K-only His-UB constructs. All experiments were independently performed in triplicate, yielding consistent results.

Since RNF25 regulates NF-κB activity independently of the activation of these upstream kinases and p65 is a core component of the NF-κB pathway 48, we hypothesized that p65 could be a ubiquitination target of RNF25. However, while RNF25 was found to bind to p65 at endogenous levels, it did not induce p65 ubiquitination (Figure 3A, S4A), nor did RNF25 knockdown affect p65 protein levels (Figure 2E, S4B), suggesting that p65 is not the direct ubiquitination target of RNF25. To identify other potential targets, we performed tandem affinity purification and mass spectrometry, which highlighted TRIP4 and LRPPRC as top candidates interacting with both RNF25 and p65 (Figure 3B). Co-immunoprecipitation (co-IP) assays and confirmed interactions at both ectopic and endogenous levels (Figure 3C, S4C). GST pull-down assays further demonstrated a direct interaction among RNF25, TRIP4, and p65 *in vitro* (Figure 3D, S4D). Ubiquitination assays revealed that RNF25 specifically catalyzes the ubiquitination of TRIP4, but not LRPPRC (Figure 3E, S4E). Notably, the catalytically inactive RNF25 mutant (2CA) failed to promote TRIP4 ubiquitination like WT RNF25 both *in vivo* and *in vitro* (Figure 3F and G). RNF25 knockdown also reduced endogenous TRIP4 ubiquitination (Figure 3H). Importantly, knockdown of TRIP4, but not LRPPRC, enhanced p65 phosphorylation and overall NF-κB activity in SW839 and OS-RC-2 cells (Figure 3I-K, S4F and G). Moreover, TRIP4 knockdown reversed the reduction in p65 phosphorylation and the suppression of NF-κB activity caused by RNF25 knockdown (Figure 3I-K). Intriguingly, TRIP4 was found to interact predominantly with p65 and p50, but not with components of the upstream IKK complex (Figure 3L), in line with our observation that RNF25 regulates NF-κB activity independently of upstream kinases. Together, these findings suggest that TRIP4 is the direct ubiquitination target of RNF25 and is essential for NF-κB activation.

While RNF25 promotes the ubiquitination of TRIP4, RNF25 knockdown did not affect TRIP4 protein levels (Figure 3M, S4H). To investigate the nature of RNF25-mediated poly-ubiquitination of TRIP4, we expressed wild-type ubiquitin and single lysine residue-only or exclusive mutants in 293T cells and revealed that RNF25 specifically enhanced K27-linked poly-ubiquitination of TRIP4 (Figure 3N, S4I). These findings indicate that TRIP4 is a direct, non-degradative ubiquitination target of RNF25 and plays a critical role in NF-κB activation.

### RNF25 promotes poly-ubiquitination of TRIP4 at lysine-135, disrupting its interaction with p65

**Figure 4:**
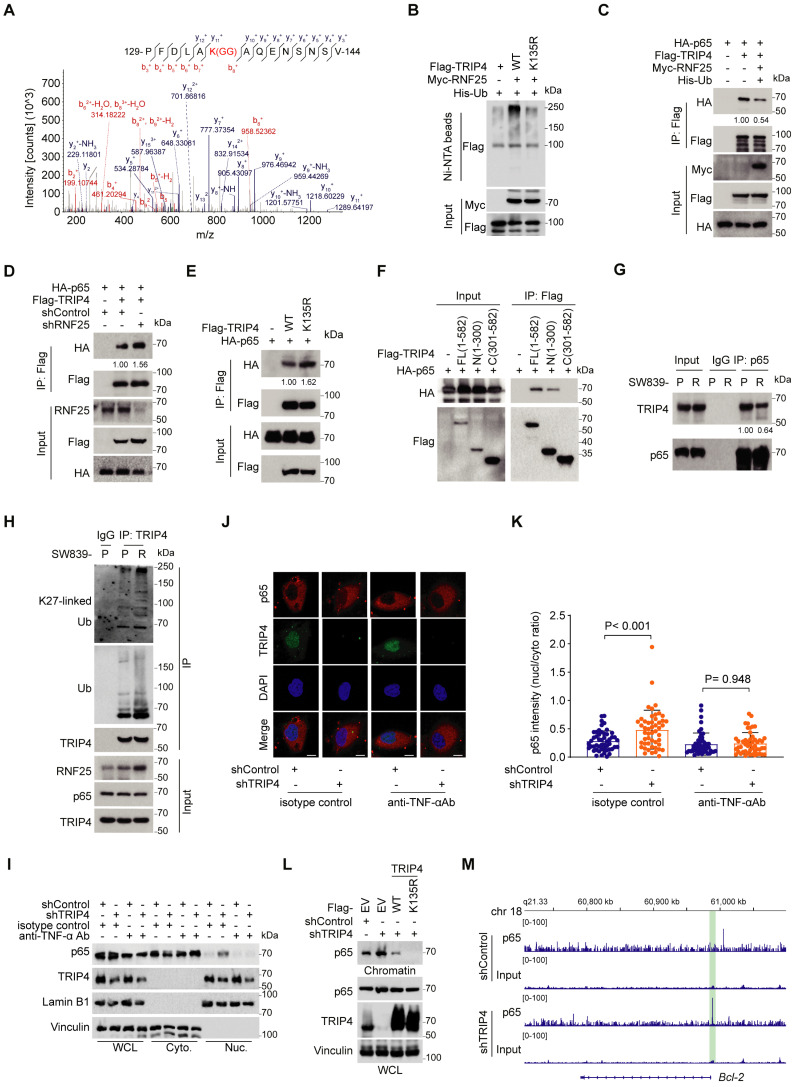
** RNF25 promotes poly-ubiquitination of TRIP4 at lysine-135, disrupting its interaction with p65. A** Mass spectrometry identified lysine 135 of TRIP4 as a potential ubiquitination site. **B** Western blot analysis of input samples and Ni-NTA affinity precipitates from 293T cells transfected with Myc-RNF25, His-UB, and Flag-TRIP4 constructs. **C** Western blot analysis of input samples and IP derived from 293T cells transfected with indicated plasmids. **D** Western blot analysis of input samples and IP derived from control and RNF25-knockdown 293T cells transfected with HA-p65 and Flag-TRIP4. **E** Western blot analysis of input samples and IP derived from 293T cells transfected with HA-p65 and Flag-TRIP4 constructs. **F** Western blot analysis of input samples and IP derived from 293T cells transfected with HA-p65 and Flag-TRIP4 constructs. **G** Western blot analysis of input samples and anti-p65 immunoprecipitates derived from parental and resistant SW839 cells. **H** Western blot analysis of input samples and anti-TRIP4 immunoprecipitates derived from parental and resistant SW839 cells. **I** Western blot analysis of WCL, cytosolic (Cyto.) and nuclear (Nuc.) fractions from control and TRIP4-knockdown SW839 cells treated with either an isotype control or a TNF-α neutralizing antibody. **J, K** Representative images of p65 immunofluorescence in control and TRIP4-knockdown SW839 cells treated with either an isotype control or a TNF-α neutralizing antibody (**J**). The nuclear-to-cytoplasmic fluorescence ratio of p65 was quantified for each cell (**K**) (mean ± SD, n = 50, one-way ANOVA). Scale bar, 10 μm. **L** Western blot analysis of WCL and chromatin binding proteins derived from control and TRIP4-knockdown SW839 cells infected with the indicated viral constructs. **M** ChIP-seq analysis of p65 occupancy at the *Bcl-2* gene locus in control and TRIP4-knockdown SW839 cells. All experiments were independently performed in triplicate, yielding consistent results.

We further investigated the ubiquitination site of RNF25-mediated poly-ubiquitination of TRIP4. Mass spectrometry of Flag-tagged TRIP4 co-expressed with RNF25 and ubiquitin in 293T cells identified Lys135 as the ubiquitination site in TRIP4 (Figure 4A). Mutation of Lys135 abolished RNF25-induced TRIP4 poly-ubiquitination (Figure 4B), confirming that RNF25 promotes poly-ubiquitination at Lys135 of TRIP4. Since TRIP4 interact with both RNF25 and p65, and knockdown of TRIP4 had opposite effects to RNF25 knockdown on p65 phosphorylation (Figure 3I-K), we hypothesized that RNF25-mediated TRIP4 ubiquitination is key for p65-dependent NF-κB activation. To address this, we examined whether TRIP4 ubiquitination influences its interaction with p65. Co-IP data showed that transient expression of RNF25 reduced the interaction between ectopically expressed TRIP4 and p65 (Figure 4C). Conversely, RNF25 knockdown enhanced TRIP4-p65 binding (Figure 4D). Mutation of Lys135 in TRIP4 strengthened its binding to p65 (Figure 4E). Moreover, Co-IP with TRIP4 and p65 truncations confirmed that the N-terminal (1-300 aa) of TRIP4 and full-length p65 are essential for their interaction (Figure 4F, S5A), suggesting that Lys135 ubiquitination of TRIP4 may disrupt its interaction with p65. We further examined the endogenous TRIP4-p65 interaction in both parental and apoptosis-resistant SW839 cells and found that the interaction was reduced in resistant cells compared to parental cells, coinciding with elevated levels of endogenous K27-linked ubiquitination of TRIP4 (Figure 4G and H). These data indicate that the K27-linked ubiquitination of TRIP4 impairs its interaction with p65.

We next investigated the functional consequence of the TRIP4-p65 interaction. Given that TRIP4 is a direct ubiquitination target of RNF25 and is essential for NF-κB activation independently of upstream kinases, we sought to determine whether TRIP4 affects p65 nuclear translocation. To rule out the possibility that NF-κB activation resulted from feedback-induced upstream kinase activity, cells were treated with a TNF-α-neutralizing antibody. Both cellular fractionation and immunofluorescence staining displayed similar results that TRIP4 knockdown had little effect on p65 nuclear translocation under TNF-α-neutralizing conditions, indicating that TRIP4 does not directly influence p65 nuclear translocation (Figure 4I-K, S5B-D). Additionally, we found that TRIP4 is exclusively localized in the nucleus (Figure 4J, S5C), and its knockdown did not significantly affect the interaction between p65 and IκBα (Figure S5E), further supporting the conclusion that TRIP4 does not regulate the cytoplasmic retention of p65 through IκBα.

We then explored whether RNF25-mediated ubiquitination of TRIP4 at Lys135 influences p65 chromatin binding. Chromatin fractionation assays revealed that TRIP4 knockdown enhanced p65 association with chromatin, an effect reversed by re-expression of WT TRIP4 (Figure 4L). In contrast, expression of the K135R TRIP4 mutant, which binds more strongly to p65, completely abolished p65 association with chromatin. Moreover, ChIP-seq analysis using a p65 antibody in control and TRIP4 knockdown SW839 cells revealed increased p65 binding at the *Bcl-2* gene locus following TRIP4 knockdown (Figure 4M). These findings suggest that RNF25-mediated polyubiquitination of TRIP4 at Lys135 facilitates its dissociation from p65, thereby promoting p65 binding to chromatin and enhancing NF-κB activation.

### Ubiquitination TRIP4 at Lys135 facilitates NF-κB activation and apoptosis resistance

**Figure 5:**
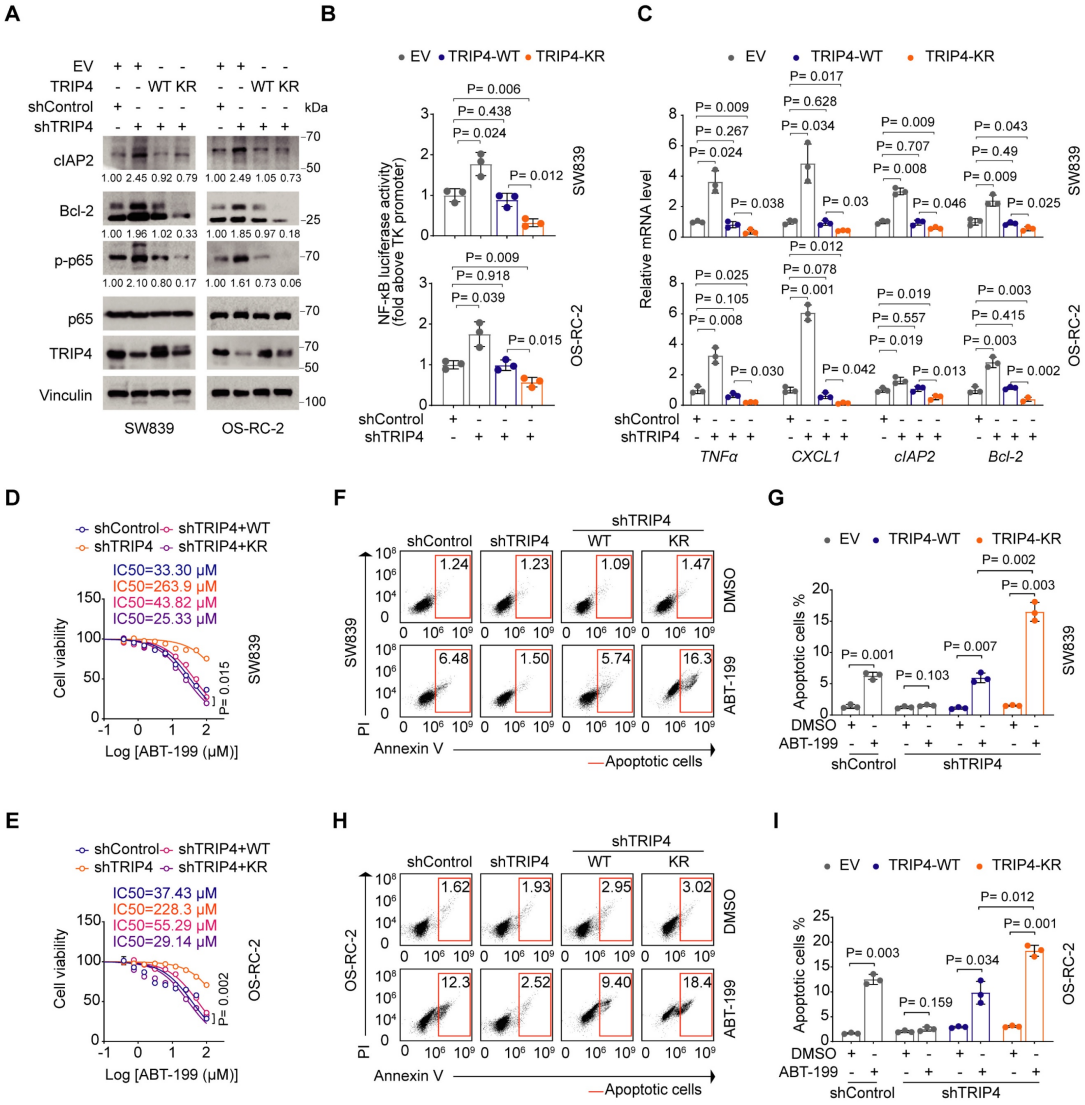
** Ubiquitination TRIP4 at Lys135 facilitates NF-κB activation and apoptosis resistance. A** Western blot analysis of WCL derived from control and TRIP4-knockdown SW839 and OS-RC-2 cells infected with the indicated viral constructs. Relative protein levels of p-p65, Bcl-2 and cIAP2 are shown. **B**,** C** Dual luciferase reporter assay (**B**) and RT‒qPCR (**C**) were performed in control and TRIP4-knockdown SW839 (**top**) and OS-RC-2 (**bottom**) cells infected with the indicated viral constructs (mean ± SD, n = 3, two-tailed unpaired Student's *t*-test).**D, E** Dose-response survival curves of control and TRIP4-knockdown SW839 (**D**) and OS-RC-2 (**E**) cells infected with the indicated viral constructs exposed to increasing concentrations of ABT-199 (mean ± SEM, n = 3). **F, G** Annexin V/7-AAD-FC analysis of control and TRIP4-knockdown SW839 cells infected with the indicated viral constructs treated with DMSO or ABT-199 (20 µM) for 24 hours (**F**), with quantification data shown in (**G**) (mean ± SD, n = 3, two-tailed unpaired Student's *t*-test). **H, I** Annexin V/7-AAD-FC analysis of control and TRIP4-knockdown OS-RC-2 cells infected with the indicated viral constructs treated with DMSO or ABT-199 (20 µM) for 24 hours (**H**), with quantification data shown in (**I**) (mean ± SD, n = 3, two-tailed unpaired Student's *t*-test). All experiments were independently performed in triplicate, yielding consistent results.

We then sought to evaluate the function of TRIP4 ubiquitination at Lys135 in apoptosis resistance. While TRIP4 knockdown resulted in increased phosphorylation of p65, leading to heightened NF-κB activation, rescue expression of K135R mutant abolished this effect more substantially than WT TRIP4. Similar trends were observed with the NF-κB downstream targets cIAP2 and Bcl-2 (Figure 5A-C). Furthermore, compared to TRIP4 WT, the K135R mutant notably reduced the IC50 value for ABT-199 in SW839 and OS-RC-2 cells (Figure 5D and E), correlating with a greater proportion of apoptotic cells in the K135R mutant group (Figure 5F-I). Overall, our results suggest that the ubiquitination of TRIP4 at Lys135 plays a crucial role in facilitating NF-κB activation and promoting apoptosis resistance.

### BAY11-7082 directly binds RNF25, reversing RNF25-mediated apoptosis suppression

**Figure 6:**
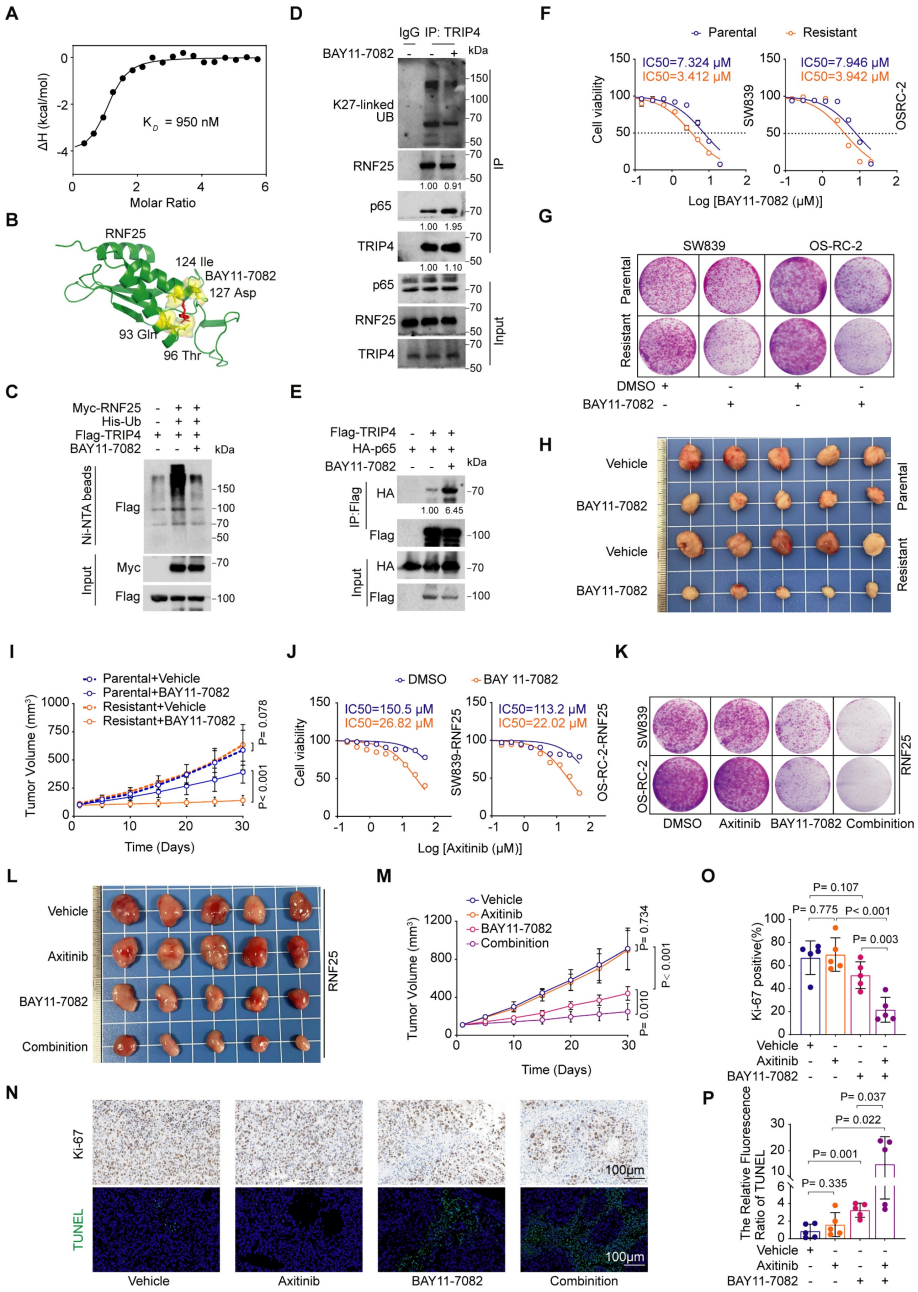
** BAY11-7082 directly binds RNF25, reversing RNF25-mediated apoptosis suppression. A** Binding affinity measured by isothermal titration calorimetry (ITC) between RNF25 and BAY11-7082. **B** Schematic illustration of the predicted potential binding site of BAY 11-7082 on RNF25, based on structural modeling with AlphaFold 3. **C** Western blot analysis of WCL and Ni-NTA affinity precipitates derived from 293T cells transfected with Flag-TRIP4, His-UB and Myc-RNF25, followed by treatment with BAY11-7082 (1 µM) for 24 hours. **D** Western blot analysis of input samples and anti-TRIP4 immunoprecipitates derived from SW839 cells treated with DMSO or BAY11-7082. **E** Western blot analysis of input samples and IP derived from 293T cells transfected with HA-p65 and Flag-TRIP4, treated with DMSO or BAY11-7082. **F** Dose-response survival curves of parental and resistant SW839 (**left**) and OS-RC-2 (**right**) cells exposed to increasing concentrations of BAY11-7082 (mean ± SEM, n = 3). **G** Colony formation assays were performed in parental and resistant SW839 and OS-RC-2 cells treated with DMSO or BAY11-7082 (0.1 µM). **H, I** Parental and resistant SW839 cells were injected subcutaneously into the right flank of nude mice. Mice received intraperitoneal injections of either vehicle or BAY11-7082 (10 mg/kg) daily for 14 days. Tumors were harvested and photographed on day 30 (**H**). Tumor volume was measured at the indicated time points (**I**) (mean ± SD, n = 5, two-sided two-way ANOVA, n = 5). **J** Dose-response survival curves of RNF25-overexpressing SW839 (**left**) and OS-RC-2 (**right**) cells treated with DMSO or BAY11-7082 (1 µM), exposed to increasing concentrations of axitinib (mean ± SEM, n = 3). **K** Colony formation assays were performed in RNF25-overexpressing SW839 and OS-RC-2 cells treated with DMSO, axitinib, BAY11-7082, or a combination of axitinib and BAY11-7082. **L, M** RNF25-overexpressing SW839 cells were injected subcutaneously into the right flank of nude mice. BAY 11-7082 (10 mg/kg) was administered via intraperitoneal injection, and axitinib (30 mg/kg) was given via oral gavage, with both treatments lasting for 14 days. Tumors were harvested and photographed on day 30 (**L**). Tumor volume was measured at the indicated time points (**M**) (mean ± SD, n = 5, two-sided two-way ANOVA). **N** Histopathological analysis of the excised tumors from each treatment group using Ki-67 staining and TUNEL assay. Scale bar: 100 μm. **O, P** Quantification of the proportion of Ki-67-positive cells (**O**) and the relative TUNEL fluorescence intensity (**P**) (mean ± SD, n = 5, two-tailed unpaired Student's *t*-test). All experiments were independently performed in triplicate, yielding consistent results.

Our small-molecule inhibitor screening revealed that while RNF25 overexpression conferred resistance to most drugs, RNF25-overexpressing cells showed heightened sensitivity to the NF-κB inhibitor BAY11-7082 (Figure 1J, S6A-C), a multi-target small molecule best known for inhibiting IκB kinase (IKK), thereby blocking activation of the NF-κB signaling pathway 49, 50. However, we found that knockdown RNF25 showed less increase of BAY11-7082 sensitivity in normal SW839 and OS-RC-2 cells (Figure S6D-F). Given the role of RNF25 in NF-κB activation, this sensitivity suggests additional underlying mechanisms. To resolve this dichotomy, we tested whether BAY11-7082 directly targets RNF25. Isothermal titration calorimetry (ITC) using purified full-length RNF25 demonstrated direct binding between BAY11-7082 and RNF25 with a moderate affinity (K*_D_* = 950 nM) but not other two additional commercially available NF-κB inhibitors (Figure 6A, S6G and H). Dose survival and colony formation assays in SW839 and OS-RC-2 cells showed that, unlike BAY11-7082, the other two compounds did not induce increased sensitivity in RNF25-overexpressing cells (Figure S6I-N). AlphaFold3 51, 52 structural predictions further mapped the interaction interface to critical residues (Gln93, Thr96, Ile124, Asp127) within a putative functional pocket of RNF25 (Figure 6B). We then validated the inhibitory effect of BAY11-7082 on RNF25 function. BAY11-7082 treatment significantly reduced p65 phosphorylation (Figure S7A), consistent with the observed decrease in NF-κB activity (Figure S7B), while RNF25 protein levels remained unchanged. However, BAY11-7082 treatment reduced both ectopic RNF25-mediated TRIP4 ubiquitination (Figure 6C) and endogenous K27-linked ubiquitination of TRIP4 (Figure 6D). Correspondingly, an increased interaction between TRIP4 and p65 was observed following BAY11-7082 treatment (Figure 6E). These findings identify BAY11-7082 as a novel RNF25-binding compound and suggest its hypersensitivity in RNF25-high cells may result from dual suppression of RNF25 and NF-κB signaling.

To explore BAY11-7082's effect on RNF25-mediated apoptosis resistance, we treated apoptosis-resistant SW839 and OS-RC-2 cell lines with BAY11-7082, observing lower IC50 values and reduced colony formation compared to parental cells (Figure 6F and G, S7C). *In vivo*, BAY11-7082 more effectively suppressed apoptosis-resistant tumor growth than parental tumors (Figure 6H and I). We then sought to determine whether BAY11-7082 could overcome RNF25-mediated axitinib resistance. To this end, we analyzed cell lysates and performed flow cytometry on cells treated with axitinib and BAY11-7082, either individually or in combination (Figure S7D-F). The results revealed that co-treatment with BAY11-7082 and axitinib induced significantly higher levels of apoptosis compared to either treatment alone. We next evaluated the therapeutic efficacy of this combination strategy both *in vitro* and *in vivo*. In RNF25-overexpressing SW839 and OS-RC-2 cells, BAY11-7082 treatment significantly reduced the IC50 of axitinib compared to DMSO controls (Figure 6J) and enhanced its inhibitory effect on colony formation when used in combination (Figure 6K, S7G). Furthermore, CompuSyn analysis 53 confirmed the combination of BAY11-7082 and axitinib synergistically reduced the viability of SW839 and OS-RC-2 cells (Figure S7H). *In vivo*, the addition of BAY11-7082 to axitinib significantly enhanced tumor growth inhibition (Figure 6L and M) and apoptosis in RNF25-overexpressing SW839 xenografts (Figure 6N-P). These findings demonstrate that BAY11-7082, by targeting both NF-κB and RNF25, augments axitinib's therapeutic efficacy, offering a promising strategy to overcome axitinib resistance.

## Discussion

Apoptosis is primarily regulated through pro-apoptotic mechanisms, acting as a natural barrier to cancer development 6, 9, 54. The intrinsic pathway is triggered by cellular stressors like DNA damage and reactive oxygen species (ROS), leading to the release of cytochrome c from the mitochondria and subsequent caspase activation and cell death 2. In contrast, anti-apoptotic signals inhibit this process and promote cell survival. Anti-apoptotic proteins such as Bcl-2 and Bcl-xL prevent mitochondrial disruption by sequestering pro-apoptotic members, while inhibitor of apoptosis proteins (IAPs) directly inhibit caspases, allowing tumor cells to evade programmed cell death 2. A significant player in the regulation of anti-apoptotic signals is the NF-κB pathway, which, upon activation by inflammatory cytokines and growth factors, upregulates anti-apoptotic genes like Bcl-2, Bcl-xL, and XIAP 21, 22, 25. In the present study, we identified a *de novo* anti-apoptotic factor RNF25, which is essential for NF-κB activation. By developing apoptosis-resistant SW839 and OS-RC-2 RCC cell lines through continuous exposure to the selective Bcl-2 inhibitor ABT-199, we observed that RNF25 was overexpressed in these resistant cells. RNF25 is highly expressed across a range of tumor types (Figure 1I).

Pan-cancer analyses from cBioPortal reveal no recurrent mutations, deletions, or amplifications of RNF25 across most malignancies. However, in the Kidney Renal Clear Cell Carcinoma (TCGA, Nature 2013) cohort 55, individual cases have been reported to carry mutations such as *X96_splice*, *L224P*, and *P86L*. Although widespread genomic alterations are not observed, our finding may still hold functional significance of RNF25 in tumor therapy. We further elucidated the underlying mechanism: RNF25 promotes the non-degradative ubiquitination of TRIP4 at Lys135, facilitating the release of p65 from TRIP4 binding. This action activates NF-κB signaling and leads to the upregulation of anti-apoptotic genes, including *cIAP2* and *Bcl-2*.

Targeting apoptotic pathways in tumor cells has been recognized as a potent anti-cancer strategy, as overcoming apoptosis resistance significantly enhances clinical responses and reduces relapse risks 8, 56. Currently, most approved therapies induce cell death indirectly, such as through the inhibition of growth factor signaling, kinase, mTOR, the proteasome, or histone deacetylases 56. In contrast, Bcl-2-targeting agents represent a class of FDA-approved drugs that directly engage the intrinsic apoptotic pathway and have shown substantial clinical benefit 31, 57, 58. Among them, ABT-199, a selective Bcl-2 inhibitor, has demonstrated potent efficacy in preclinical models of CLL and NHL characterized by Bcl-2 overexpression, without inducing thrombocytopenia 31. Nevertheless, developing effective therapies that target cell death pathways remains challenging due to the multitude of therapeutic targets and resistance mechanisms present in different tumor types 56. Our findings indicate that RNF25 is overexpressed in various cancer types and contributes to resistance against various small molecule inhibitors, particularly apoptosis-inducing agents like gemcitabine and docetaxel, suggesting a previously unexplored mechanism underlying drug resistance. Notably, we identified the NF-κB inhibitor BAY11-7082 as a dual-function compound that directly binds RNF25 (K*_D_* = 950 nM) and abrogates its ability to suppress apoptosis and drive therapeutic resistance. This polypharmacological activity—simultaneously targeting NF-κB signaling and RNF25—reveals a synergistic mechanism to overcome apoptosis resistance in RNF25-high malignancies, positioning BAY11-7082 as a template for novel combinatorial therapies.

## Conclusion

In conclusion, our study reveals that the E3 ubiquitin ligase RNF25 plays a critical role in promoting NF-κB-driven resistance to apoptosis in cancer cells, facilitating escape from various targeted treatments. RNF25 interacts with TRIP4, inducing its non-degradative ubiquitination at lysine 135, which prevents TRIP4 from associating with p65. This action frees p65 to trigger NF-κB signaling, boosting the expression of survival factors like *cIAP2* and *Bcl-2*. Additionally, we show that BAY11-7082, an NF-κB signaling pathway inhibitor, directly binds RNF25, counteracting its anti-apoptotic effects and restoring susceptibility to cell death. Our study highlights RNF25 as a druggable regulator of therapy resistance via NF-κB signaling modulation and supports BAY11-7082 as a potential therapeutic approach to overcome apoptosis resistance in cancer.

## Supplementary Material

Supplementary figures and tables.

## Abbreviations

RCC: Renal cell carcinoma
CLL: Chronic lymphocytic leukemia
NHL: Non-Hodgkin lymphoma
MS: Mass spectrometry
TCGA: The Cancer Genome Atlas
TUNEL: Terminal deoxynucleotidyl transferase dUTP nick end labeling
GSEA: Gene set enrichment analysis
RT-qPCR: Reverse transcription-quantitative polymerase chain reaction
IC50: Half-maximal inhibitory concentration
WT: Wild-type
IP: Immunoprecipitation
GST: Glutathione S-transferase
ROS: Reactive oxygen species
IAPs: Inhibitor of apoptosis proteins
FC: Flow cytometry
ITC: Isothermal titration calorimetry
ChIP-seq: Chromatin Immunoprecipitation Sequencing
P: Parental
R: Resistant
EV: Empty vector
