# Supplementary material for: BAY11-7082 Targets RNF25 to Reverse TRIP4 Ubiquitination-dependent NF-κB Activation and Apoptosis Resistance in Renal Cell Carcinoma
Source: Int J Biol Sci. 2025 Jul 4;21(10):4410–27. doi: 10.7150/ijbs.115032 (PMC12320230; doi:10.7150/ijbs.115032)
Supplement: Supplementary file 1 — Supplementary figures and tables. [file ijbsv21p4410s1.pdf]

Supplementary Materials

Supplementary Figures

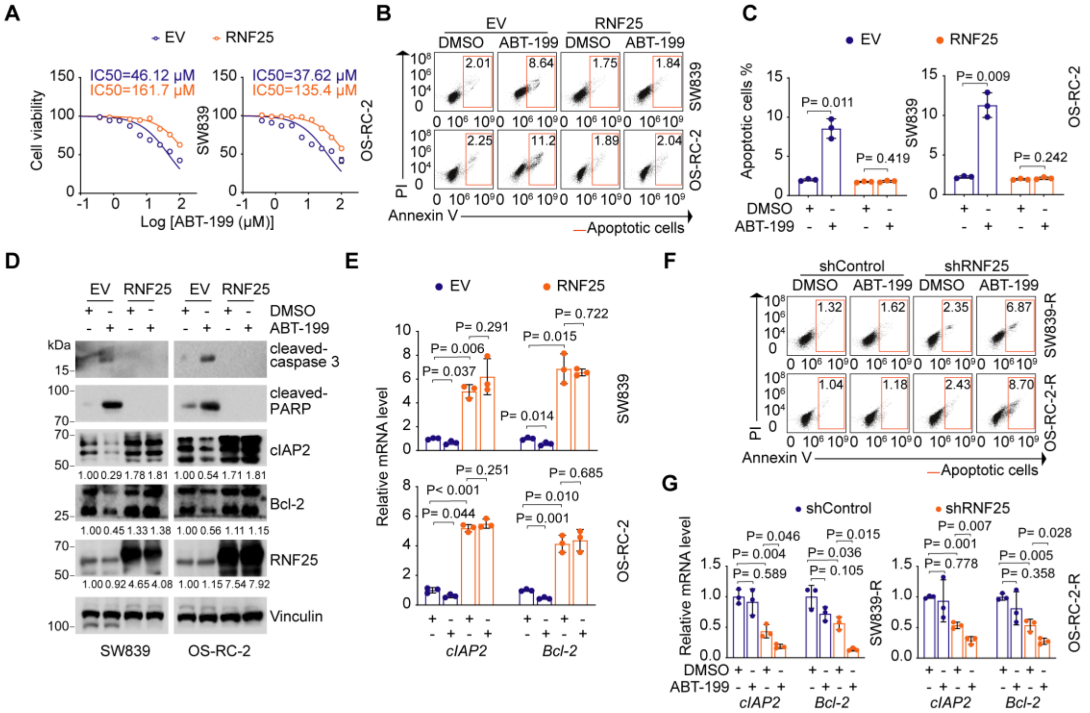

Figure S1. Identification of RNF25 as a negative regulator of apoptosis. Related to Figure 1. **A** Dose-response survival curves of control and RNF25-overexpressing SW839 (**left**) and OS-RC-2 (**right**) cells exposed to increasing concentrations of ABT-199 (mean±SEM, n = 3). **B, C** Annexin V/7-AAD-FC analysis of control and RNF25-overexpressing SW839 and OS-RC-2 cells treated with DMSO or ABT-199 (20 µM) for 24 hours (**B**), with quantification data shown in (**C**) (mean±SD, n = 3, two-tailed unpaired Student's *t*-test). **D** Western blot analysis of WCL derived from control and RNF25-overexpressing SW839 and OS-RC-2 cells treated with DMSO or ABT-199 (20 µM) for 24 hours. Relative protein levels of RNF25, cIAP2 and Bcl-2 are shown. **E** RT–qPCR analysis of control and RNF25-overexpressing SW839 (**top**) and OS-RC-2 cells (**bottom**) treated with DMSO or ABT-199 (20 µM) for 24 hours (mean±SD, n = 3, two-tailed unpaired Student's *t*-test). **F** Annexin V/7-AAD-FC analysis of control and RNF25-knockdown SW839-R and OS-RC-2-R cells treated with DMSO or ABT-199 (20 µM) for 24 hours. **G** RT–qPCR analysis of control and RNF25-knockdown SW839-R cells (**left**) and OS-RC-2-R cells (**right**) treated with DMSO or ABT-199 (20 µM) for 24 hours (mean±SD, n = 3, two-tailed unpaired

Student's *t*-test). All experiments were independently performed in triplicate, yielding consistent results.

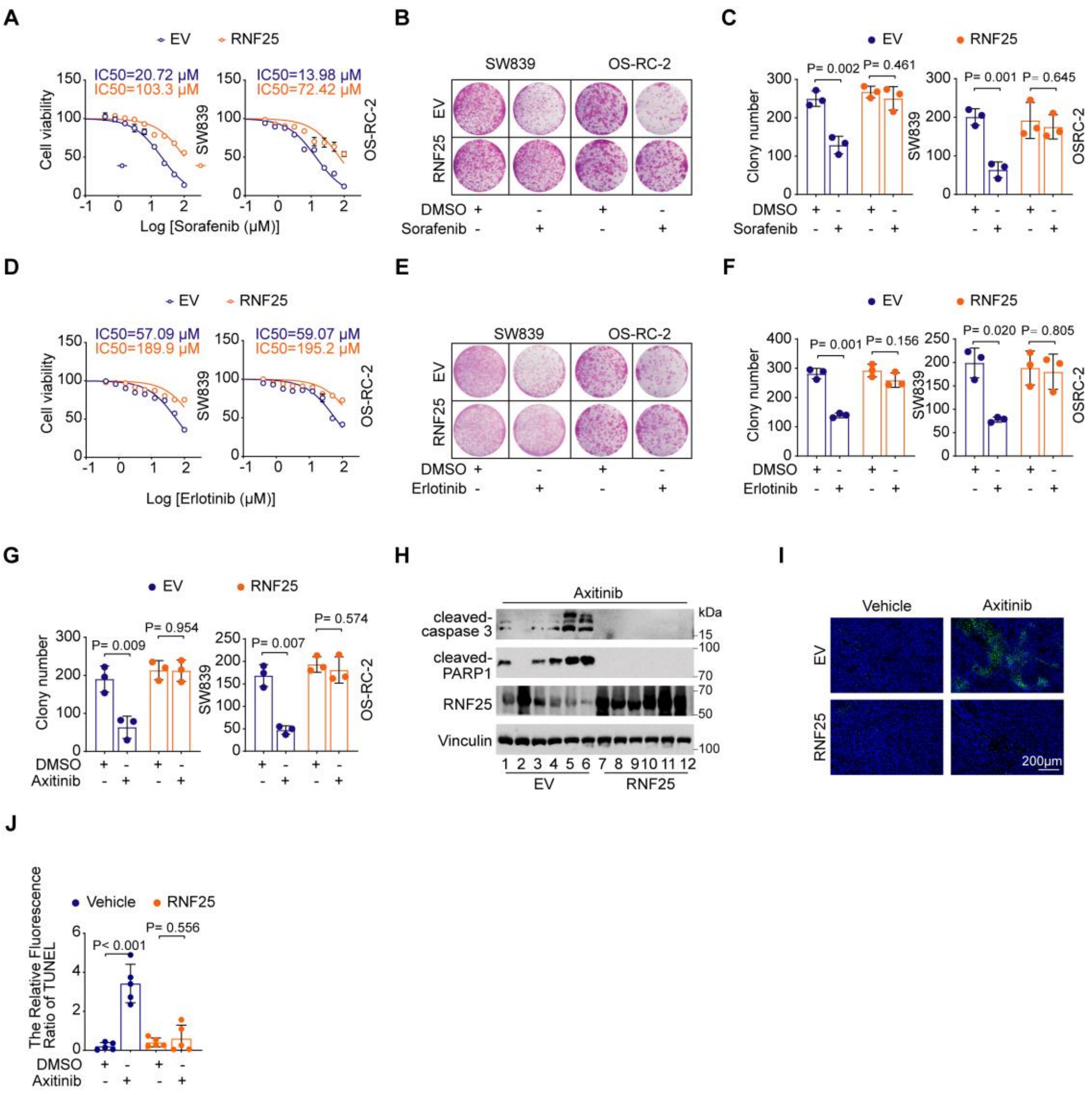

Figure S2. RNF25 overexpression induces tyrosine kinase inhibitor resistance in RCC. Related to Figure 1. **A** Dose-response survival curves of control and RNF25-overexpressing SW839 (**left**) and OS-RC-2 (**right**) cells exposed to increasing concentrations of sorafenib (mean±SEM, n = 3). **B, C** Colony formation assays were performed in control and RNF25-overexpressing SW839 and OS-RC-2 cells treated with DMSO or sorafenib (1 μM). Representative colonies are shown in (**B**) with quantification data shown in (**C**) (mean±SD, n = 3, two-tailed unpaired Student's *t*-test). **D** Dose-response survival curves of control and RNF25-overexpressing SW839 (**left**) and OS-RC-2 (**right**) cells exposed to increasing concentrations of erlotinib (mean±SEM, n = 3). **E, F** Colony formation assays were performed in control and

RNF25-overexpressing SW839 and OS-RC-2 cells treated with DMSO or erlotinib (10 μM). Representative colonies are shown in (**E**) with quantification data shown in (**F**) (mean±SD, n = 3, two-tailed unpaired Student's *t*-test). **G** Quantification data of Colony formation assays in control and RNF25-overexpressing SW839 and OS-RC-2 cells treated with DMSO or axitinib (5 μM) (mean±SD, n = 3, two-tailed unpaired Student's *t*-test). **H** Western blot analysis of WCL derived from control and RNF25-overexpressing tumors in Fig 1M. **I, J** Representative images of TUNEL assays in SW839 tumors from each treatment group are shown in Fig. 1M (**I**). Scale bar: 200 μm. Quantification of relative TUNEL fluorescence intensity is presented in (**J**) (mean ± SD, n = 5; two-tailed unpaired Student's t-test). All experiments were independently performed in triplicate, yielding consistent results.

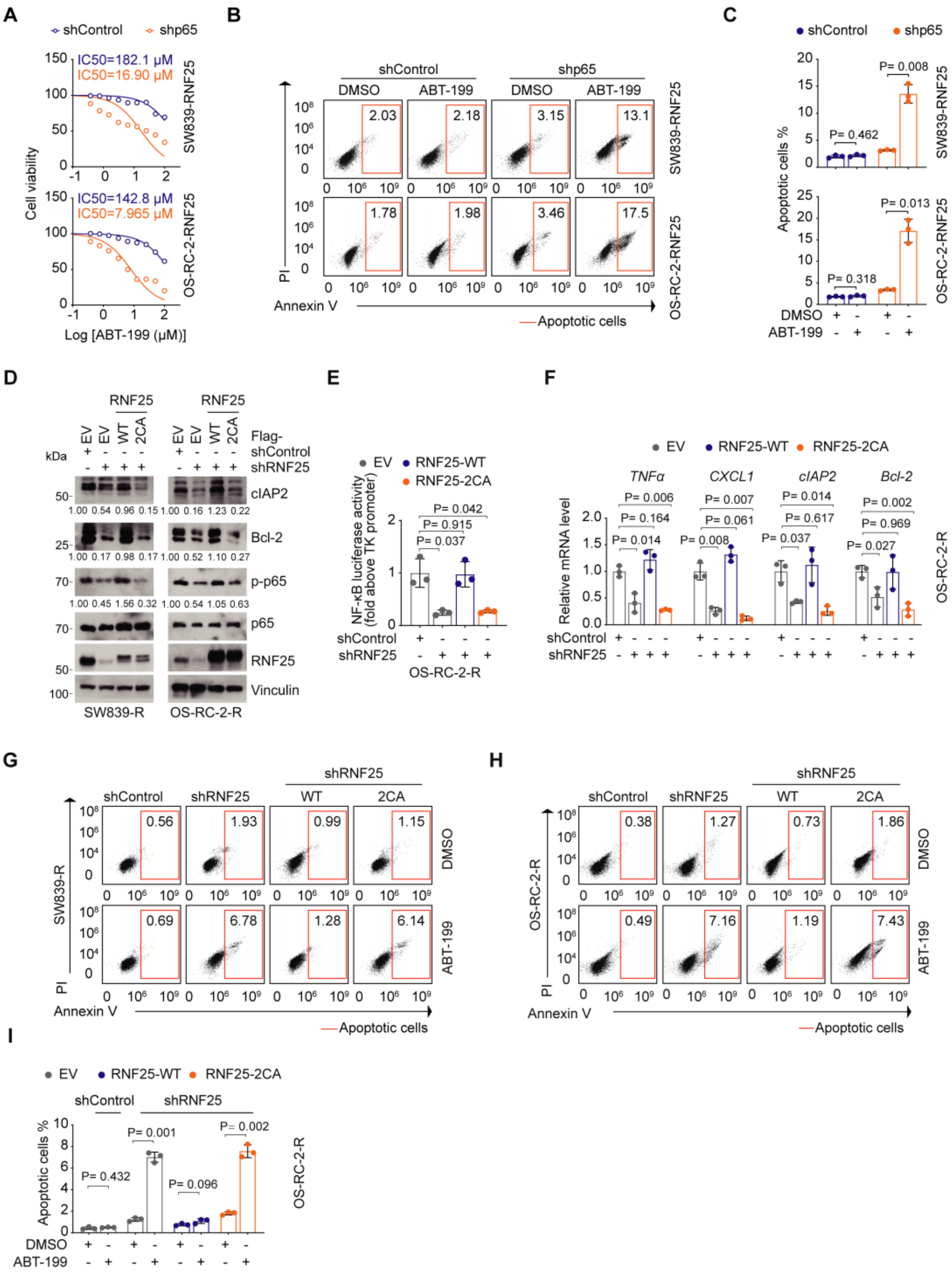

Figure S3. NF-κB activation is necessary for RNF25-mediated anti-apoptosis. Related to Figure 2. A Dose-response survival curves of control and p65-knockdown RNF25-overexpressing SW839 (**left**) and OS-RC-2 (**right**) cells exposed to increasing concentrations of ABT-199 (mean ± SEM, n = 3). **B, C** Annexin V/7-AAD-FC analysis of p65-knockdown RNF25-overexpressing SW839 and OS-RC-2 cells treated with DMSO or ABT-199 (20 μM) for 24 hours (**B**), with quantification data shown in (**C**) (mean±SD, n = 3, two-tailed unpaired Student's *t*-test). **D** Western

blot analysis of WCL derived from RNF25-knockdown SW839-R and OS-RC-2-R cells infected with the indicated viral constructs. Relative protein levels of p-p65, Bcl-2and cIAP2 are shown. **E, F** Dual luciferase reporter assay (**E**) and RT–qPCR (**F**) were performed in RNF25-knockdown OS-RC-2-R cells infected with the indicated viral constructs (mean ± SD, n = 3, two-tailed unpaired Student's *t*-test). **G** Annexin V/7-AAD-FC analysis of RNF25-knockdown SW839-R cells infected with the indicated viral constructs treated with DMSO or ABT-199 (20 μM) for 24 hours. **H, I** Annexin V/7-AAD-FC analysis of RNF25-knockdown OS-RC-2-R cells infected with the indicated viral constructs treated with DMSO or ABT-199 (20 μM) for 24 hours (**H**), with quantification data shown in (**I**) (mean±SD, n = 3, two-tailed unpaired Student's *t*-test). All experiments were independently performed in triplicate, yielding consistent results.

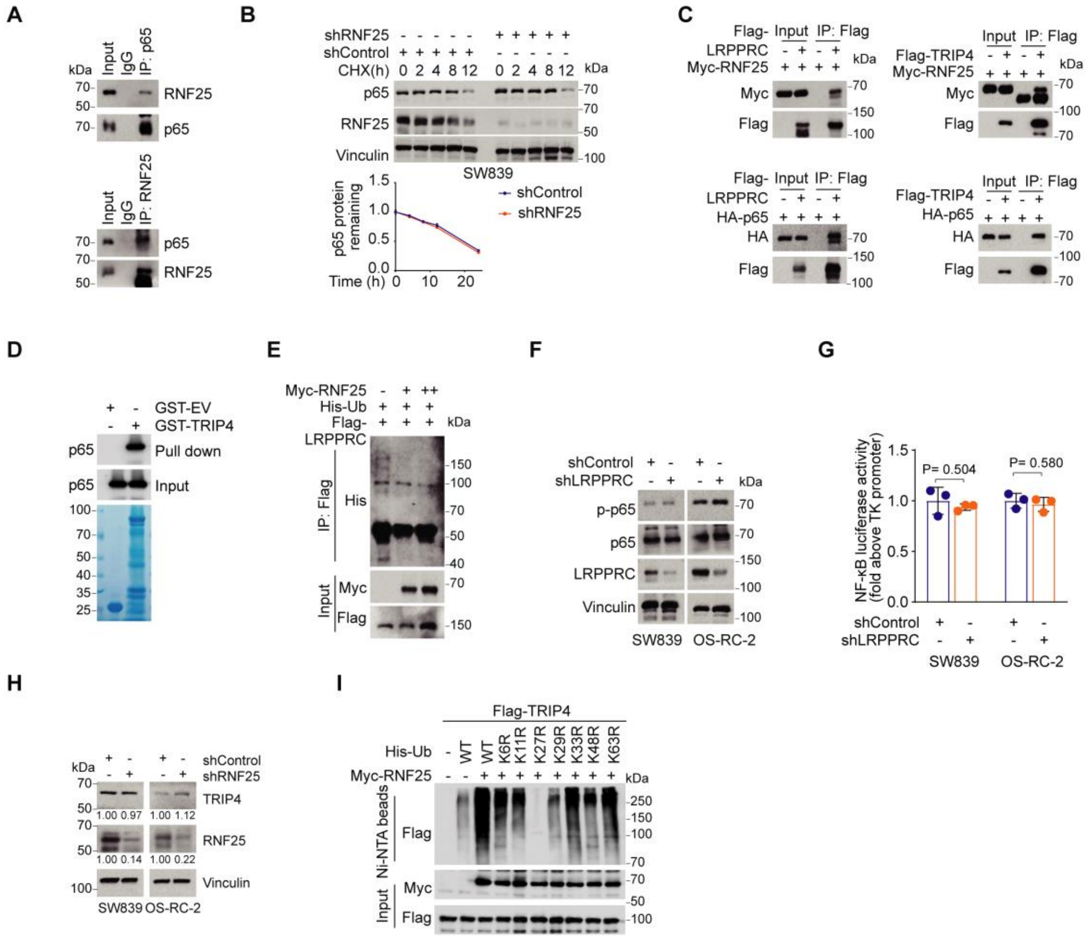

Figure S4. TRIP4 is a ubiquitination target of RNF25 essential for NF-κB activation. Related to Figure 3. A Western blot analysis of input samples and anti-RNF25 or anti-p65 immunoprecipitates derived from SW839 cells. **B** Western blot

analysis of control and RNF25-knockdown SW839 cells treated with 200 μg/μl CHX at the indicated time points (**top**), and protein bands were quantified (**bottom**). **C** Western blot analysis of input samples and IP derived from 293T cells transfected with indicated plasmids. **D** Western blot analysis of p65 proteins in SW839 cells pulled down by GST-EV or GST- TRIP4 recombinant proteins. **E** Western blot analysis of input samples and IP derived from 293T cells transfected with Flag-LRPPRC, His-UB and increasing amounts of Myc-RNF25. **F** Western blot analysis of WCL derived from control and LRPPRC-knockdown SW839 and OS-RC-2 cells. **G** Dual luciferase reporter assays were performed in control and LRPPRC-knockdown SW839 and OS-RC-2 cells (mean ± SD, n = 3, two-tailed unpaired Student's *t*-test). **H** Western blot analysis of WCL derived from control and RNF25-knockdown SW839 and OS-RC-2 cells. Relative protein levels of RNF25 and TRIP4 are shown. **I** Western blot analysis of WCL and Ni-NTA affinity precipitates derived from 293T cells transfected with Flag-TRIP4 and the indicated Myc-RNF25, WT, or KR His-UB constructs. All experiments were independently performed in triplicate, yielding consistent results.

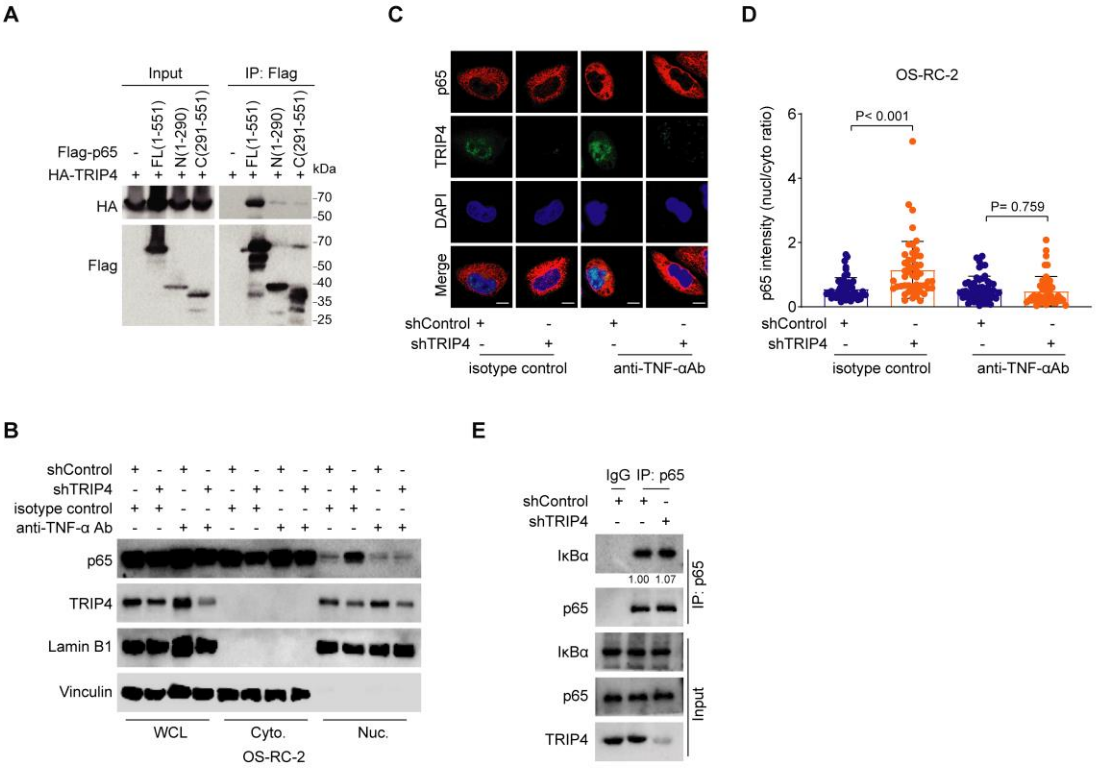

Figure S5. RNF25 promotes poly-ubiquitination of TRIP4 at lysine-135, disrupting its interaction with p65. Related to Figure 4. A Western blot analysis of input samples and IP derived from 293T cells transfected with HA- TRIP4 and Flag-p65 constructs. **B** Western blot analysis of WCL, cytosolic (Cyto.) and nuclear (Nuc.) fractions from control and TRIP4-knockdown OS-RC-2 cells treated with either an isotype control or a TNF-α neutralizing antibody. **C, D** Representative images of p65 immunofluorescence in control and TRIP4-knockdown OS-RC-2 cells treated with either an isotype control or a TNF-α neutralizing antibody (**C**). The nuclear-to-cytoplasmic fluorescence ratio of p65 was quantified for each cell (**D**) (mean ± SD, n = 50, one-way ANOVA). Scale bar, 10 µm. **E** Western blot analysis of input samples and anti-p65 immunoprecipitates derived from control and TRIP4-knockdown SW839 cells. All experiments were independently performed in triplicate, yielding consistent results.

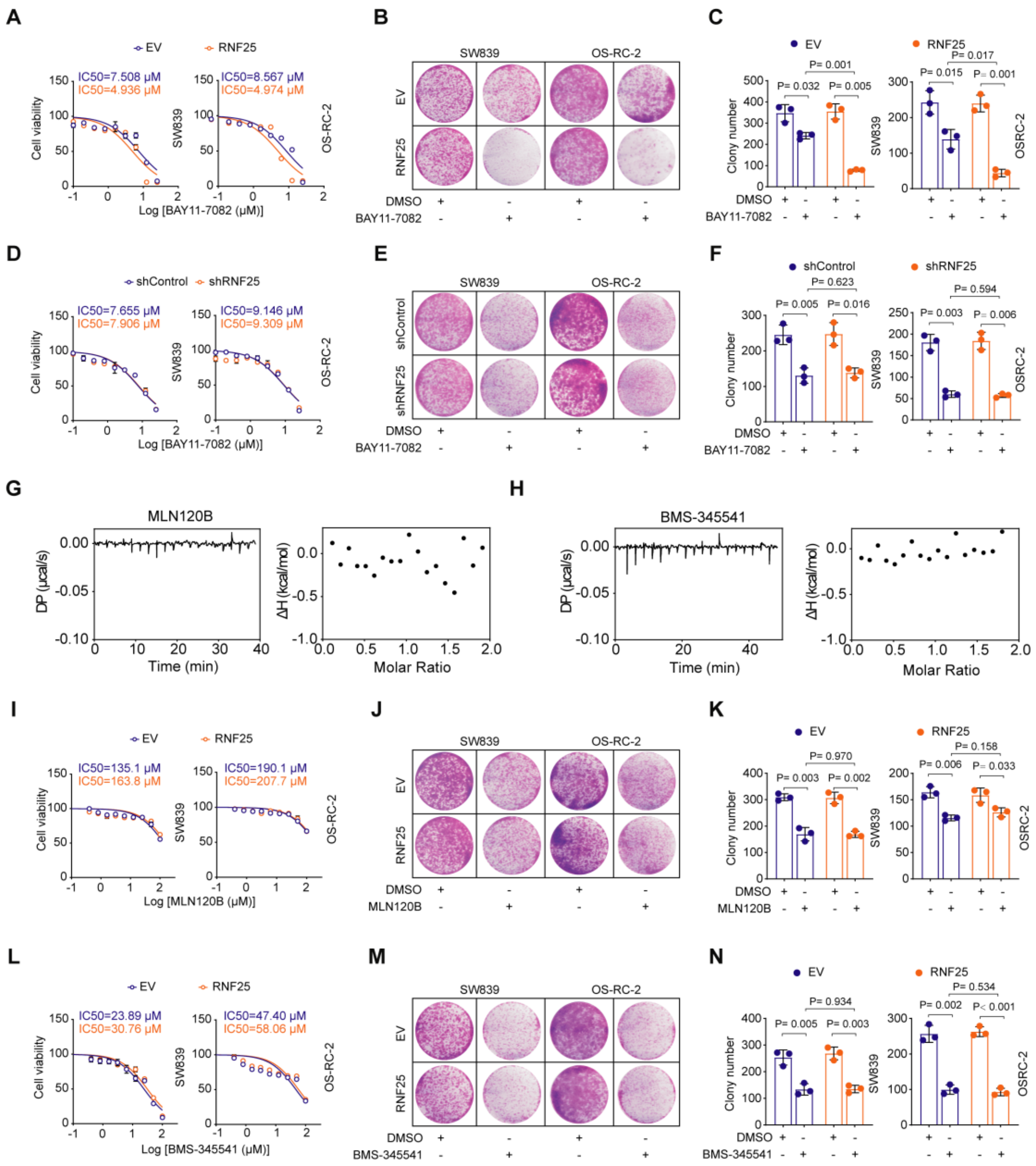

Figure S6. BAY11-7082 directly binds RNF25, reversing RNF25-mediated apoptosis suppression. Related to Figure 6. A Dose-response survival curves of RNF25-overexpressing SW839 (**left**) and OS-RC-2 (**right**) cells exposed to increasing concentrations of BAY11-7082 (mean±SEM, n = 3). **B, C** Colony formation assays were performed in control and RNF25-overexpressing SW839 and OS-RC-2 cells treated with DMSO or BAY11-7082 (0.1 μM). Representative colonies are shown in (**B**), with quantification data shown in (**C**) (mean±SD, n = 3, two-tailed unpaired Student's *t*-test). **D** Dose-response survival curves of control and RNF25-knockdown SW839 (**left**) and OS-RC-2 (**right**) cells exposed to increasing concentrations of BAY11-7082 (mean±SEM, n = 3). **E, F** Colony formation assays were performed in control and RNF25-knockdown SW839 and OS-RC-2 cells treated with DMSO or BAY11-7082 (0.1 μM). Representative colonies are shown in (**E**), with quantification data shown in (**F**) (mean±SD, n = 3, two-tailed unpaired Student's *t*-test). **G, H**

Binding affinity measured by isothermal titration calorimetry (ITC) between RNF25, MLN120B (**G**), and BMS-345541 (**H**). **I** Dose-response survival curves of RNF25-overexpressing SW839 (**left**) and OS-RC-2 (**right**) cells exposed to increasing concentrations of MLN120B (mean±SEM, n = 3). **J, K** Colony formation assays were performed in control and RNF25-overexpressing SW839 and OS-RC-2 cells treated with DMSO or MLN120B (10 μM). Representative colonies are shown in (**J**), with quantification data shown in (**K**) (mean±SD, n = 3, two-tailed unpaired Student's *t*-test). **L** Dose-response survival curves of RNF25-overexpressing SW839 (**left**) and OS-RC-2 (**right**) cells exposed to increasing concentrations of BMS-345541 (mean± SEM, n = 3). **M, N** Colony formation assays were performed in control and RNF25-overexpressing SW839 and OS-RC-2 cells treated with DMSO or BMS-345541 (5 μM). Representative colonies are shown in (**M**), with quantification data shown in (**N**) (mean±SD, n = 3, two-tailed unpaired Student's *t*-test). All experiments were independently performed in triplicate, yielding consistent results.

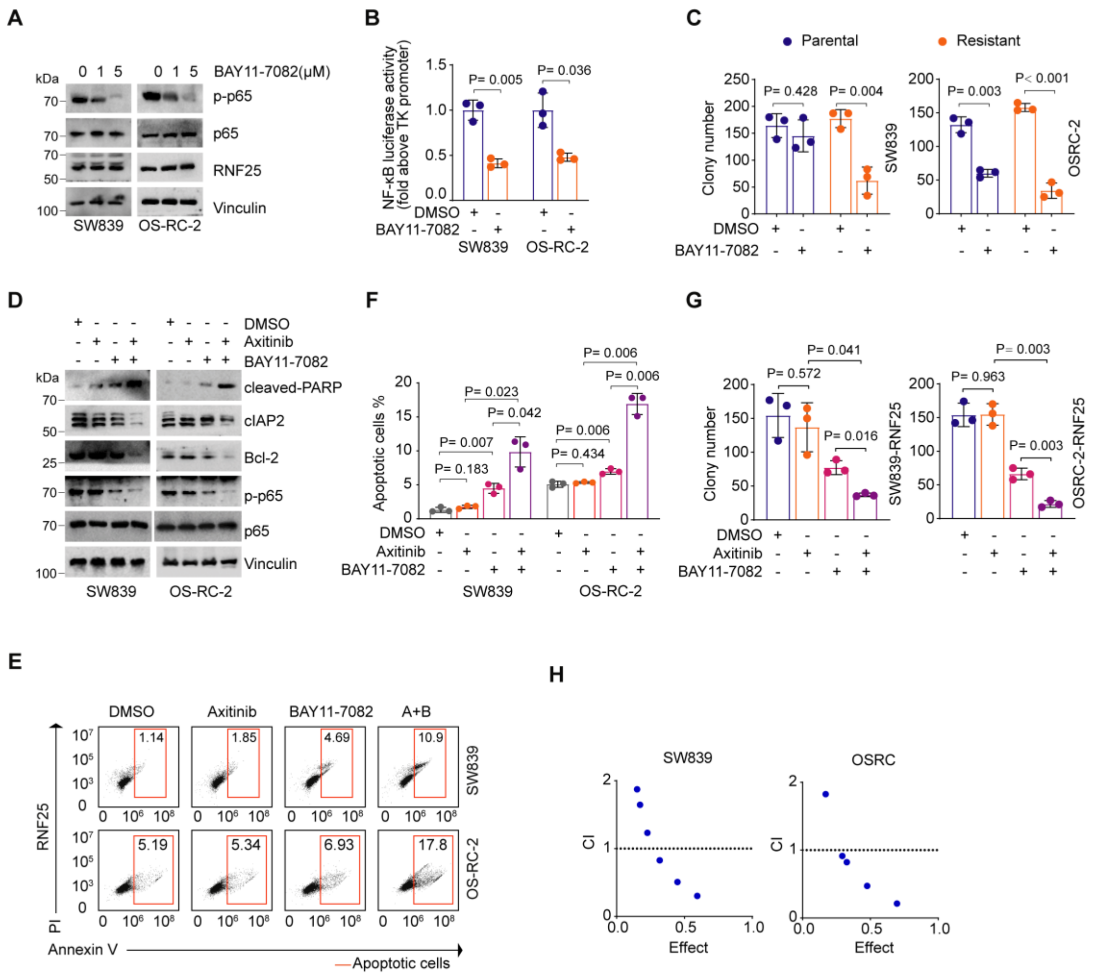

Figure S7. Combination of BAY11-7082 and axitinib as a strategy to combat axitinib resistance. Related to Figure 6. A Western blot analysis of WCL derived from SW839 and OS-RC-2 cells treated with DMSO or BAY11-7082 for 24 hours. **B** Dual luciferase reporter assays were performed in SW839 and OS-RC-2 cells treated

with DMSO or BAY11-7082 (1 μM) for 24 hours (mean ± SD, n = 3, two-tailed unpaired Student's *t*-test). **C** Quantification of colony formation assays in parental and resistant SW839 and OS-RC-2 cells treated with DMSO or BAY11-7082 (0.1 μM) (mean±SD, n = 3, two-tailed unpaired Student's *t*-test). **D** Western blot analysis of WCL derived from SW839 and OS-RC-2 cells treated with DMSO, axitinib (20 μM), BAY11-7082 (1 μM), or a combination of axitinib and BAY11-7082 for 24 hours. **E, F** Annexin V/7-AAD-FC analysis of SW839 and OS-RC-2 cells treated with DMSO, axitinib (20 μM), BAY11-7082 (1 μM), or a combination of axitinib and BAY11-7082 for 24 hours (**E**), with quantification data shown in (**F**) (mean±SD, n = 3, two-tailed unpaired Student's *t*-test). **G** Quantification of colony formation assays in RNF25-overexpressing SW839 and OS-RC-2 cells treated with DMSO, axitinib, BAY11-7082, or a combination of axitinib and BAY11-7082 (mean±SD, n = 3, two-tailed unpaired Student's *t*-test). **H** Combination Index (CI) plot of axitinib and BAY11-7082 treatment of SW839 (left) and OS-RC-2 (right) cells. SW839 and OS-RC-2 cells were co-treated with 1 μM BAY11-7082 and different concentrations of axitinib for 24h, and the viability of the cells was measured by the MTT assay. Combination index (CI) values were calculated by the Chou and Talalay method, using the CompuSyn Software, version 1 (ComboSyn, Inc.). The data points below or above the line indicate synergistic or antagonistic drug interactions, respectively. All experiments were independently performed in triplicate, yielding consistent results.

Supplementary Tables

Table S1. **Sequences of RT-qPCR primers, Related to Figure 2, Figure 3 and Figure 5 and Figure S1 and Figure S3.**

Name	Species	Sequence of forward primer	Sequence of reverse primer
18S rRNA	Homo sapiens	GAGGTTCGAAGACGATCAGA	TCGCTCCACCAACTAAGAAC
RNF25	Homo sapiens	AGGACTGGGTCCTTCCCTCT	CTGGCCATGAGGGATGTTGT
TRIP4	Homo sapiens	GGAGATCATTCAGTACGTTTGTCA	CTCTGCAGTCGTGTCAGGTT
TNFα	Homo sapiens	GAGGCCAAGCCCTGGTATG	CGGGCCGATTGATCTCAGC
CXCL1	Homo sapiens	ATTCACCCCAAGAACATCCA	CACCAGTGAGCTTCCTCCTC
cIAP2	Homo sapiens	CCGTCAAGTTCAAGCCAGTTACCC	AGCCCATTTCCACGG CAGCA
Bcl-2	Homo sapiens	GTGGAGGAGCTCTTCAGGGA	AGGCACCCAGGGTGATGCAA

Table S2. **Antibodies**

Name	Source	Catalog Number	RRID
Rabbit polyclonal anti-RNF25	Proteintech	Cat# 24536-1-AP	RRID: AB_2879594
Rabbit polyclonal anti-TRIP4	Proteintech	Cat# 12324-1-AP	RRID: AB_10646482
Rabbit polyclonal anti-cIAP2	Proteintech	Cat# 24304-1-AP	RRID: AB_2879485
Rabbit polyclonal anti- Bcl-2	Proteintech	Cat# 12789-1-AP	RRID: AB_2227948
Rabbit polyclonal anti-Caspase 3/p17/p19	Proteintech	Cat# 19677-1-AP	RRID: AB_10733244
Rabbit polyclonal anti-Cleaved-PARP1	Zenbio	Cat# 380374;	N/A

Rabbit polyclonal anti- IκBα	Cell Signaling Technology	Cat# 4812;	RRID: AB_10694416
Rabbit monoclonal anti-Phospho-IκBα (Ser32)	Cell Signaling Technology	Cat# 2859;	N/A
Mouse monoclonal anti-p65	ABclonal	Cat# A18210;	RRID: AB_2861986
Mouse monoclonal anti-p65	Santa Cruz	Cat# sc-8008X;	RRID: AB_628017
Rabbit monoclonal anti-Phospho-NF-kB p65/RelA-S536	ABclonal	Cat# AP1294;	RRID: AB_3099756
Rabbit polyclonal anti-Phospho-IKKα/β (Ser176/180) (16A6)	Cell Signaling Technology	Cat# 2697S;	RRID: AB_2079382
Mouse monoclonal LRPPRC	Santa Cruz	Cat# sc-166178;	RRID: AB_2137453
Mouse monoclonal Vinculin	Santa Cruz	Cat# sc-73264;	RRID: AB_1131292
Rabbit monoclonal anti-Ubiquitin (linkage-specific K27)	Abcam	Cat# ab181537	RRID: AB_2713902
Rabbit monoclonal anti-Ubiquitin (E4I2J)	Cell Signaling Technology	Cat# 43124;	RRID: AB_2799235
Rabbit monoclonal anti-His-tag	Proteintech	Cat# 10001-0-AP;	RRID: AB_11232228
Anti-DDDDK-tag mAb-HRP-DirecT	MBL	Cat# M185-7;	N/A
Mouse monoclonal anti-c-Myc	Santa Cruz	Cat# sc-40;	RRID: AB_627268
Rabbit monoclonal anti-HA-tag	ABclonal	Cat# AE105;	RRID: AB_2943030

HRP Goat Anti-Rabbit IgG (H+L)	ABclonal	Cat # AS014;	N/A
HRP Goat Anti-Mouse IgG (H+L)	ABclonal	Cat # AS003;	N/A
